# Genome-Wide Patterns of Bracovirus Chromosomal Integration into Multiple Host Tissues during Parasitism

**DOI:** 10.1128/JVI.00684-21

**Published:** 2021-10-27

**Authors:** Héloïse Muller, Mohamed Amine Chebbi, Clémence Bouzar, George Périquet, Taiadjana Fortuna, Paul-André Calatayud, Bruno Le Ru, Julius Obonyo, Laure Kaiser, Jean-Michel Drezen, Elisabeth Huguet, Clément Gilbert

**Affiliations:** a Université Paris-Saclay, CNRS, IRD, UMR Évolution, Génomes, Comportement, et Écologie, Gif-sur-Yvette, France; b UMR 7261 CNRS, Institut de Recherche sur la Biologie de l'Insecte, Faculté des Sciences et Techniques, Université de Tours, Tours, France; c ViroScan3D SAS, Lyon, France; d International Centre of Insect Physiology and Ecology, Institut de Recherche pour le Développement Team, Nairobi, Kenya; Cornell University

**Keywords:** bracovirus, chromosomal integration, genomics, horizontal transfer, host-parasite relationship, parasitoid wasps, polydnavirus

## Abstract

Bracoviruses are domesticated viruses found in parasitic wasp genomes. They are composed of genes of nudiviral origin that are involved in particle production and proviral segments containing virulence genes that are necessary for parasitism success. During particle production, proviral segments are amplified and individually packaged as DNA circles in nucleocapsids. These particles are injected by parasitic wasps into host larvae together with their eggs. Bracovirus circles of two wasp species were reported to undergo chromosomal integration in parasitized host hemocytes, through a conserved sequence named the host integration motif (HIM). Here, we used bulk Illumina sequencing to survey integrations of Cotesia typhae bracovirus circles in the DNA of its host, the maize corn borer (Sesamia nonagrioides), 7 days after parasitism. First, assembly and annotation of a high-quality genome for C. typhae enabled us to characterize 27 proviral segments clustered in proviral loci. Using these data, we characterized large numbers of chromosomal integrations (from 12 to 85 events per host haploid genome) for all 16 bracovirus circles containing a HIM. Integrations were found in four S. nonagrioides tissues and in the body of a caterpillar in which parasitism had failed. The 12 remaining circles do not integrate but are maintained at high levels in host tissues. Surprisingly, we found that HIM-mediated chromosomal integration in the wasp germ line has occurred accidentally at least six times during evolution. Overall, our study furthers our understanding of wasp-host genome interactions and supports HIM-mediated chromosomal integration as a possible mechanism of horizontal transfer from wasps to their hosts.

**IMPORTANCE** Bracoviruses are endogenous domesticated viruses of parasitoid wasps that are injected together with wasp eggs into wasp host larvae during parasitism. Several studies have shown that some DNA circles packaged into bracovirus particles become integrated into host somatic genomes during parasitism, but the phenomenon has never been studied using nontargeted approaches. Here, we use bulk Illumina sequencing to systematically characterize and quantify bracovirus circle integrations that occur in four tissues of the Mediterranean corn borer (Sesamia nonagrioides) during parasitism by the Cotesia typhae wasp. Our analysis reveals that all circles containing a HIM integrate at substantial levels (from 12 to 85 integrations per host cell, in total) in all tissues, while other circles do not integrate. In addition to shedding new light on wasp-bracovirus-host interactions, our study supports HIM-mediated chromosomal integration of bracovirus as a possible source of wasp-to-host horizontal transfer, with long-term evolutionary consequences.

## INTRODUCTION

Polydnavirus genomes within parasitoid wasps (Hymenoptera) are composed of domesticated viral genes and genes of different origins involved in virulence (1–3). The domesticated viruses encode viral particles akin to gene transfer agents, which are injected during oviposition into the lepidopteran hosts of parasitoid wasps and are necessary for successful development of the wasp larvae. Polydnaviruses result from large double-stranded DNA virus endogenization events that took place during the course of parasitoid wasp evolution (1, 4–12). Polydnaviruses of the *Ichnovirus* genus identified in the genomes of certain Ichneumonidae Campopleginae and Banchinae wasps are thought to originate from closely related virus ancestors, the nature of which is still unknown but could possibly correspond to nucleocytoplasmic large DNA viruses (10). Polydnaviruses belonging to the *Bracovirus* genus result from endogenization of a nudivirus that occurred about 100 million years ago in the ancestor of the microgastroid complex of braconid wasps (7, 13, 14), a hyperdiversified monophyletic group estimated to contain at least 46,000 species (15). Once integrated in the ancestor of microgastroid wasps, the nudivirus genes were domesticated and inherited vertically in all branches of the microgastroid tree for 100 million years. All microgastroid species studied to date express nudivirus-derived bracovirus genes in specialized cells located in a region of the ovaries named the calyx. The products of these genes form viral particles containing virulence genes (2, 16). Although the evolutionary origin of virulence genes has in most cases not been uncovered, some are clearly of wasp origin (17, 18), while others derive from transposable elements (TEs) (3, 19). They are located on so-called proviral segments dispersed in multiple chromosomal regions in the wasp’s genome (13, 20); the major one, named the macrolocus, spans 2 Mb and includes two-thirds of the proviral segments. Chromosome-scale assembly of the genome of Cotesia congregata (Microgastrinae) revealed that it contains 10 proviral loci (PL), each made of 1 to 18 proviral segments (PL2, 18 segments [3]). Comparisons between *Cotesia* species and Microplitis demolitor showed that the synteny of these PL is well conserved along the phylogeny of Braconidae in ∼53 million years of evolution, suggesting strong evolutionary constraints associated with the function of the segments.

PL belong to units that are amplified in calyx cells during particle production (21). Among those amplified units (replication units [RU]), segments are excised and circularized through site-specific recombination, which involves direct repeat junctions (DRJs) located at their extremities (3, 22–26). The resulting double-stranded DNA circles are finally packaged into viral particles, which are released in the oviduct lumen and injected into the host together with the wasp’s eggs during oviposition. Once in caterpillar host cells, DNA circle-borne virulence genes are expressed (27). Interestingly, several studies using cell culture or *in vivo* models have shown that at least some circles persist in cell lines or over the entire duration of wasp development in the form of chromosomally integrated forms (26, 28–30). Using a PCR-based approach with Sanger sequencing, integration of DNA circles was shown to occur upon parasitism for 2 circles in the hemocytes of the host of the Microplitis demolitor wasp (Microgastrinae) via a motif called the host integration motif (HIM) that is conserved in all M. demolitor bracovirus (MdBV) circles (26). Another study using primer extension capture followed by high-throughput sequencing unveiled several thousand chromosomal integrations for 8 circles of Cotesia congregata in the hemocytes of its host, the tobacco hornworm Manduca sexta (30). The 8 C. congregata proviral circles surveyed in this study contain HIM and, as reported for M. demolitor, all integrations of these circles involved two motifs, called junction 1 (J1) and junction 2 (J2), located within the HIM. J1 and J2 correspond to sequences that form the extremities of the viral sequences when integrated into lepidopteran host DNA. It was further shown that, as for MdBV circles, an ∼50-bp sequence located between J1 and J2 is lost upon integration. Integration of polydnavirus circles is not limited to bracoviruses and was also recently described for ichnoviruses. This suggests that this phenomenon plays an important role in the parasitism success of parasitoid wasps, and it reveals shared characteristics in the mechanisms underlying integration of ichnoviruses and bracoviruses (31).

In this study, we used a bulk, rather than targeted, sequencing approach to investigate polydnavirus circle persistence and integration in the host-parasitoid system involving the Cotesia typhae wasp (Microgastrinae, Braconidae) and its natural host, the Mediterranean corn borer (Sesamiae nonagrioides, Noctuidae). Cotesia typhae is a recently described species among the Cotesia flavipes species complex, and it is native to eastern sub-Saharan Africa (32). In its natural environment in East Africa, it exclusively parasitizes larvae of S. nonagrioides dwelling on Typhaceae plants. It is also able to parasitize S. nonagrioides larvae in cultivated maize fields from France; therefore, it is currently being studied as a possible biocontrol agent against this major agricultural pest (33). We first report a high-quality assembly of the whole C. typhae genome based on a hybrid sequencing approach. We found that it contains 27 typical bracovirus proviral segments as well as an unexpectedly large number of circle sequences (at least 6) that were duplicated through HIM-mediated integration. We then show that integration of all HIM-containing circles occurs systematically at high levels during parasitism in all S. nonagrioides tissues, not only in hemocytes as described for M. sexta parasitized by C. congregata (30). We further demonstrate that integration is not required for the persistence of circles during parasitism, as the quantity of nonintegrated circles is similar to that of most integrated circles in all host tissues 7 days after parasitism. Interestingly, high levels of bracovirus integration were also detected in the host’s genome even when parasitism failed.

## RESULTS

### Assembly and annotation of the C. typhae genome.

The genome of C. typhae was sequenced at about 45× depth with short paired-end reads (Illumina) and 350× depth with long reads (Oxford Nanopore Technologies [ONT]) (see Table S1 in the supplemental material). The size of the preliminary short-read assembly was 183 Mb. In agreement with this, the size of the hybrid (short- and long-read) assembly was 186,662,351 bp (see Table S2). This assembly was made of only 72 scaffolds and had an *N*_50_ value of 6.81 Mb (see Table S2). It is noteworthy that the assembly nearly reached the chromosome scale with a mean of 7.2 scaffolds per chromosome, since C. typhae has 10 chromosomes per haploid genome (34).

The completeness of the assembly was assessed using Benchmarking Universal Single-Copy Orthologs (BUSCO) (35). The results revealed that 1,639 (98.9%) of 1,658 conserved insect genes were present in the final assembly (see Table S3). Assembly visualization by Blobtools (36) using taxon-annotated GC-coverage plots showed a majority assignment to the *Polydnaviridae* family (131 Mb), which is due to the presence of bracovirus sequences dispersed in the wasp genome (3); the majority of large scaffolds were identified as containing a bracovirus sequence (see Fig. S1). Our automatic annotation revealed that 58.6% of the C. typhae genome is made of TEs. The most numerous TEs are large retrotransposon derivative (LARD) and terminal inverted repeat (TIR) elements, which represent 35 and 28% of the classified TEs, respectively (see Fig. S2). We automatically annotated a total of 8,591 genes in the genome of C. typhae (see Table S4). More than 90% of the predicted genes had over 50% of their exons supported by transcriptome sequencing (RNA-seq) from the species Cotesia vestalis, the closest species for which RNA-seq data were available. Genes exhibited a mean of 5 exons per transcript (see Table S4). The joint functional annotation procedure with InterProScan (37) and BLASTP (38) enabled us to annotate 6,488 gene models (75.5%). We were also able to transfer 781 C*otesia congregata* manually curated genes (3) into the new annotation. Of note, the number of annotated genes is lower than that of C. congregata (∼14,000 genes, among which ∼12,000 were validated by C. congregata RNA-seq data), probably in part because of the divergence between C. typhae and C. vestalis RNA sequences used for annotation.

**FIG 1:**
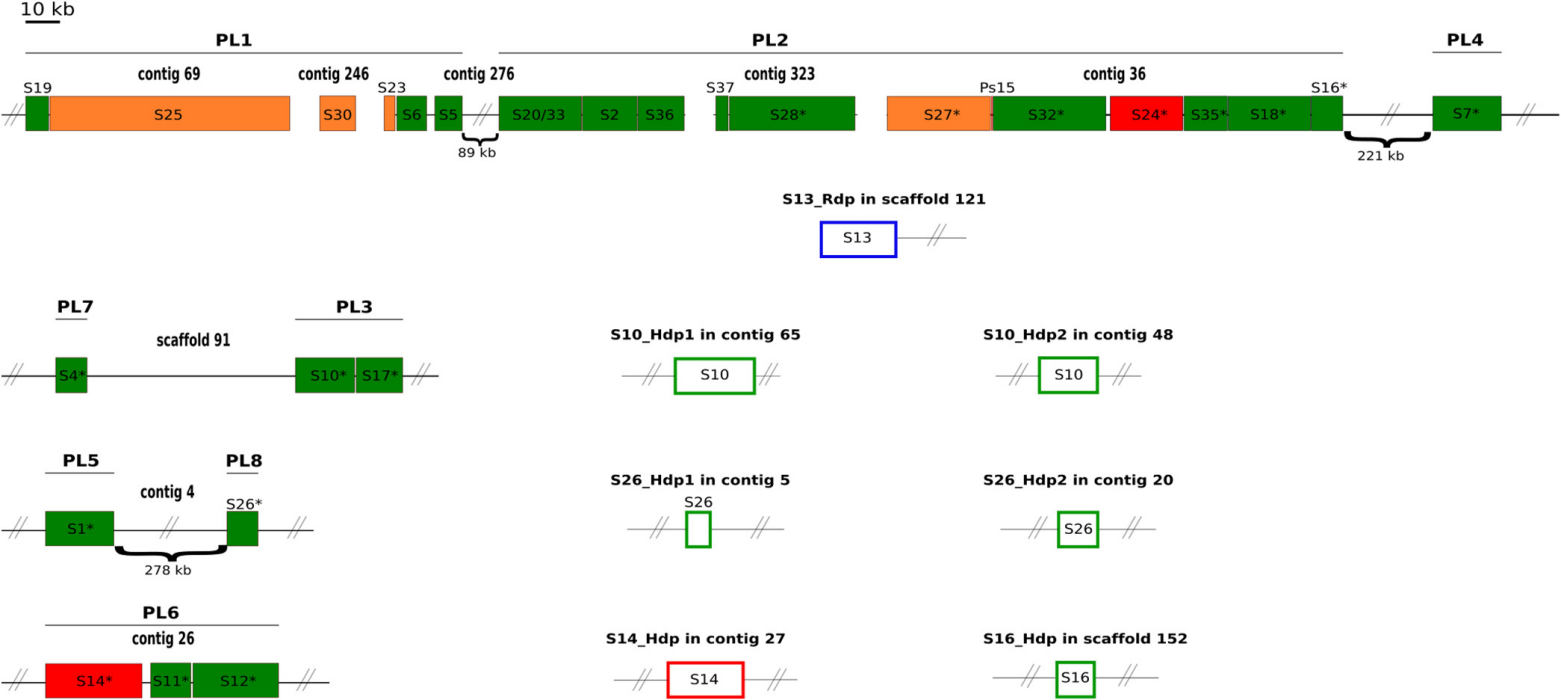
Map of CtBV proviral segments. Proviral segments are represented by filled rectangles. Segments duplicated after circularization are empty. Asterisks indicate HIM-bearing circles found to be integrated into the S. nonagrioides genome, corresponding precisely to all segments originating from the RU2.3 part of the macrolocus and isolated loci (PL3, PL4, PL5, PL6, PL7, and PL8). Each contig or scaffold in which the segments are located is indicated, and lines indicate segments that belong to the same PL. The size of the segments and the spaces between them are shown to scale, unless hash marks are present. The colors represent the quality of the annotation. Green indicates that we delimited both extremities of the segments with confidence (DRJs in proviral segments or J1 and J2 motifs in HIM-mediated duplications). Orange and red indicate that one or both extremities (see Table S5) have to be taken with caution. In the case of the orange ones, the contig was too short to identify the extremity, whereas in the case of red ones, the extremity was long enough but we were not able to find the motif. Although they are shown in green, the DRJs of S37 and S26 are truncated, probably due to sequencing or assembly issues. Blue indicates the segment duplicated after circularization by other means than HIM. In this case, there is no J1 and J2 motifs at the extremities, nor DRJ.

**FIG 2:**
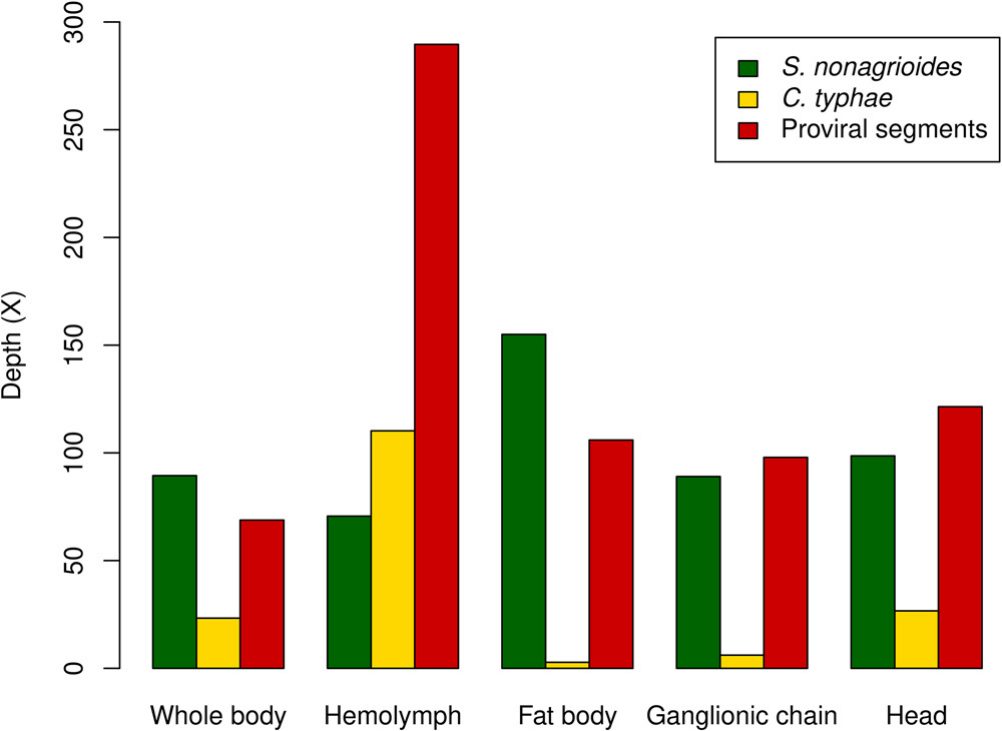
Average sequencing depths in the 5 samples. Green, yellow, and red indicate the average sequencing depths over the whole genome of S. nonagrioides, the whole genome of C. typhae, and the 27 C. typhae proviral segments, respectively.

### Annotation of C. typhae bracovirus proviral segments.

In order to annotate the proviral segments of C. typhae, we used the 26 segments of Cotesia sesamiae and the 10 segments specific to C. congregata as queries to perform similarity searches for the C. typhae genome. Twenty-seven of a total of 38 segments described in C. congregata and/or C. sesamiae were clearly identified in C. typhae (Fig. 1; also see Table S5). Among the 11 segments not found in C. typhae, 9 are specific to C. congregata, which means that they are not present in C. sesamiae either. The segment S13, which was previously found in both C. sesamiae and C. congregata, is missing in C. typhae. As in C. sesamiae, S20 and S33 are fused to form S20/33. We found 3 segments (S10, S11, and S19) that are present in C. congregata but have not been found so far in C. sesamiae. In total, we annotated 27 proviral segments in C. typhae (Fig. 1; also see Table S5).

As expected, the synteny of the segments described in other species is conserved in C. typhae (3, 20). As described in previous studies on *Glyptapanteles* and *Cotesia* species, we found a macrolocus, here gathering 18 segments, divided into two PL, PL1 and PL2 (17, 20). The 9 other segments are dispersed across six dispersed loci, from PL3 to PL8. We found that PL4 and the macrolocus are on the same scaffold, consistent with their localization on the same chromosome in C. congregata (3). We also found that PL7 and PL3 on one hand and PL5 and PL8 on the other hand are on the same chromosome as in C. congregata (3). As for C. sesamiae, we did not identify PL10 in C. typhae, suggesting that this PL is specific to C. congregata and is recent. While PL9 is present in both C. congregata (2 proviral segments) and C. sesamiae (1 proviral segment), we did not find it in C. typhae.

### Proviral segment characteristics and HIM identification.

HIMs were previously described in 12 of 36 segments in C. congregata (30). These HIMs were used here as queries to perform BLASTN searches for C. typhae segments (see Data File S2 in the supplemental material). HIMs were found at their expected homologous loci in C. typhae for all except 1 segment, S15. The lack of HIM in S15 is likely due to the fact that this segment is undergoing degradation in C. typhae. Indeed, in contrast to C. congregata, in which S15 is 8,700 bp and contains 5 genes, this segment is residual in both C. sesamiae and C. typhae, being 700 and 300 bp, respectively, and containing no gene (see below). In a second step of the analysis, we aligned the 11 HIMs identified in C. typhae against the 5 other C. typhae segments for which we found integrations in S. nonagrioides (S18, S24, S27, S28, and S32; see below). We were able to find HIM in all of these additional segments (see Data File S2). To note, our annotation of HIMs in C. typhae segments allowed us to refine the boundaries of the HIM for C. congregata S11 (see Data File S2).

Interestingly, our annotation identified seven other bracoviral sequences dispersed in the wasp genome. They share high levels of similarity with viral sequences but do not follow the organization of proviral segments with DRJs at their extremities. Without DRJs at their extremities, these segments cannot form circles and are thus not functional (see “Persistence of nonintegrated circles 7 days postoviposition” for more details). Five of these segments are clearly flanked by the J1 and J2 motifs, which normally lie within the HIM, itself located internally to the proviral segments (Fig. 1). Another segment has one-half an HIM (containing the J1 motif) at one extremity, but the contig is too short to identify the presence of the second half (containing the J2 motif) at the other extremity. Given that the structure of these 6 segments is identical to that observed after integration in the host genome (26, 30), we concluded that, as observed for 3 segments in C. sesamiae (39), these 6 C. typhae segments (namely, S10_Hdp1, S10_Hdp2, S14_Hdp, S16_Hdp, S26_Hdp1, and S26_Hdp2) originate from HIM-mediated duplication (Hdp). The 6 Hdp segments show between 97.13% and 98.9% similarity to their parental segment, suggesting that they result from relatively recent duplication events. The structure of the last nonproviral segment is atypical. It is highly similar to C. sesamiae segment 13 but it is not flanked by DRJs or HIMs. However, it possesses a single internal DRJ, presumably resulting from circularization of its parental segment via recombination of the 5′ DRJ and the 3′ DRJ. The presence of a large flanking sequence indicates that it is present as inserted into the wasp genome and not as a circle or an intermediate amplification form. Thus, we conclude that this segment is a rearranged duplication (Rdp) of S13 (S13_Rdp) that, in contrast to the duplications described above, was not mediated by HIM. To note, we were not able to find the parental segment of S13_Rdp. An explanation might be that S13 was lost after being duplicated in C. typhae. A second more plausible explanation might be that S13 is actually present in C. typhae but has not been sequenced/assembled. In this regard, according to the synteny of segments in other *Cotesia* species, S13 was expected to lie between S36 and S37 but S36 and S37 lie at the extremity of 2 different contigs (Fig. 1). In addition, sequencing depth data also suggest the presence of S13 in C. typhae (see “Persistence of nonintegrated circles 7 days postoviposition”). In this case, C. typhae would have 28 proviral segments in total. It is also noteworthy that we identified 3 other segments that we considered potentially resulting from assembly errors. These segments are highly similar to segments S1, S14, and S20/33 and thus could be real duplications of these segments. However, the contigs on which they lie (contig_14, contig_294, and contig_143) are short and do not contain any other wasp sequence (i.e., the segments are partial and not flanked by any other wasp sequence). Therefore, we decided not to include them in the annotation.

### Annotation of HIM-mediated duplications in other *Cotesia* species.

The finding of 6 HIM-mediated duplications of bracoviral segments was striking, given the absence of such duplications in high-quality genomes of M. demolitor and C. congregata (3, 26). To assess whether this feature is specific to C. typhae, we searched for HIM-mediated duplications in all other available *Cotesia* genomes (C. sesamiae, C. flavipes, Cotesia rubecula, C. vestalis, and Cotesia glomerata). The highly fragmented nature of these additional genomes prevented us from reaching a high level of confidence in the annotation of all segments. Therefore, the results of this search should be considered preliminary. Of the 6 HIM-mediated duplications, we were able to investigate whether they are shared by other *Cotesia* species for 5 of them (S16_Hdp, S10_Hdp1, S10_Hdp2, S26_Hdp1, and S26_Hdp2). Indeed, our approach relies on both J1 and J2 being clearly annotated. We identified 4 segments and 1 segment orthologous to these 5 duplications in C. sesamiae and C. flavipes, respectively. More precisely, C. sesamiae shares S10_Hdp1, S10_Hdp2, S26_Hdp1, and S26_Hdp2, whereas C. flavipes shares only S26_Hdp1. C. typhae, C. sesamiae, and C. flavipes form a monophyletic clade sister to the three other *Cotesia* species included in our search (3). In this clade, C. typhae is more closely related to C. sesamiae than to C. flavipes. These phylogenetic relationships imply that segment S26 underwent a first HIM-mediated duplication in the ancestor of the three species. Regarding the duplication of S26 and the two of S10, they occurred prior to the split between C. typhae and C. sesamiae and may even be older, as we cannot draw a conclusion about their absence in the fragmented genome of C. flavipes. We were not able to find any orthologous HIM-mediated duplications in the other *Cotesia* wasps, and we could identify only 2 candidate *de novo* HIM-mediated duplications, both in C. sesamiae. Those involve the parental segments S11 and S18.

### Sequencing depth and coverage of the genomes of C. typhae and S. nonagrioides.

We assessed the amount of host versus parasitoid DNA we sequenced in the 5 samples of parasitized S. nonagrioides (heads, hemocytes, fat body, ganglionic chain, and whole body) by separately mapping trimmed reads to the genomes of S. nonagrioides and C. typhae. We obtained a total of 335 million to 595 million trimmed reads, depending on the sample, which covered 97.9% to 99.3% of the 1,021-Mbp S. nonagrioides genome. The average sequencing depth along the S. nonagrioides genome varied between 71× and 155×, depending on the sample (Fig. 2). The percentage of reads mapping to the S. nonagrioides genome varied from 73.05% in the hemocytes to 91.94% in the fat body. Mirroring this variation, between 20.89% and 0.31% of the reads mapped to the C. typhae genome in the hemocytes and in the fat body, respectively. Thus, the vast majority of reads (92.57% to 93.94%) mapped either onto the genome of S. nonagrioides or onto that of C. typhae. The proportion of the C. typhae genome covered by the reads was high (94% to 99%) in 4 of the samples, while it dropped to 41% in the fat body. Importantly, the average coverage was higher on proviral segments (68.8× to 289.6×, depending on the sample) than on the rest of the genome (2.8× to 110.2×, depending on the sample) in all samples (Fig. 2). This is consistent with the presence of a greater proportion of integrated and/or nonintegrated wasp bracoviral circles versus other wasp genomic regions in our DNA extracts.

### HIM-mediated integration of C. typhae bracovirus DNA circles into the S. nonagrioides genome.

**FIG 3:**
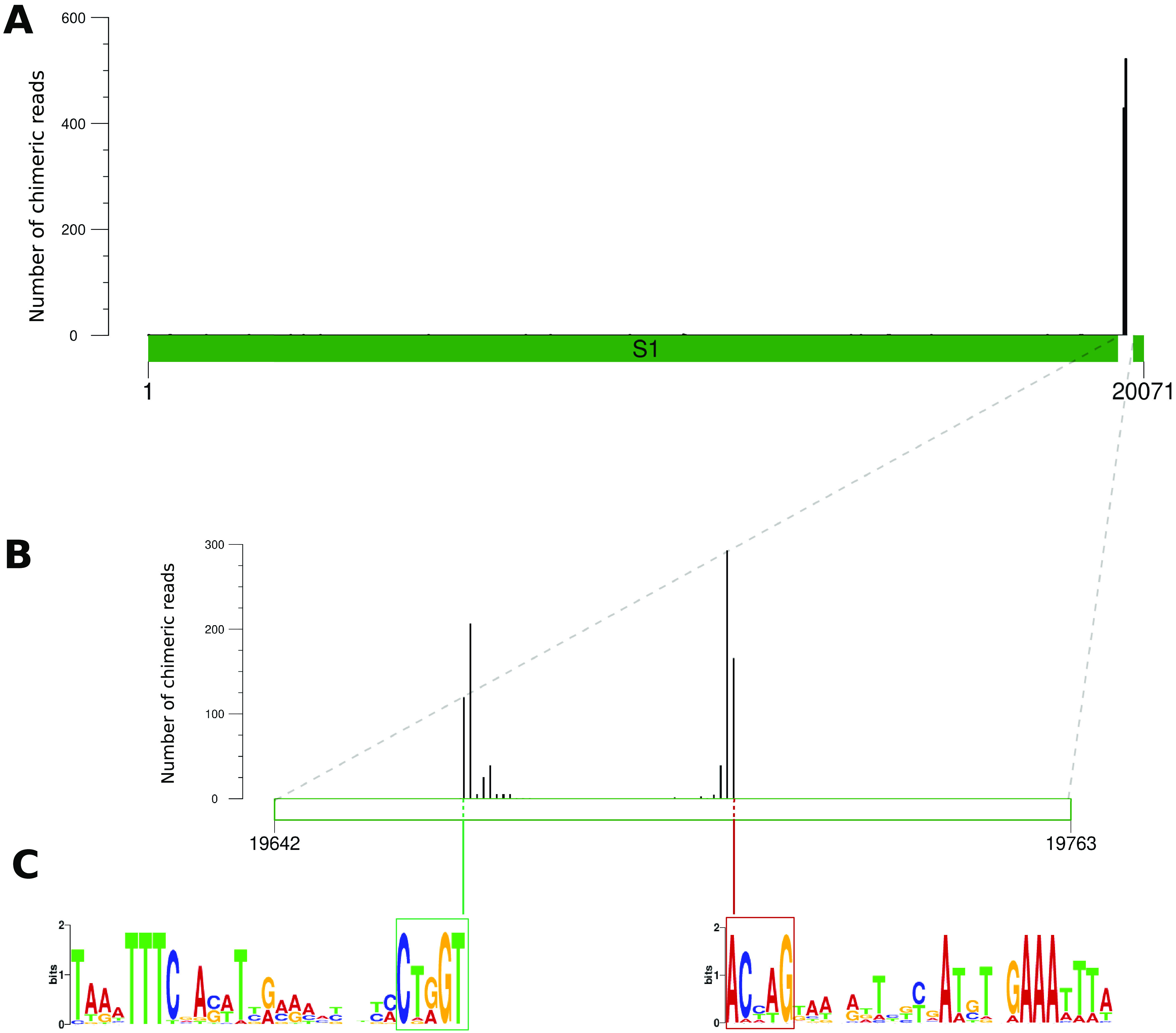
Map of chimeric reads indicating HIM-mediated chromosomal integration of segment 1. (A) Number of chimeric reads along segment 1 in hemocytes, oriented from the 5′ DRJ to the 3′ DRJ. The white portion represents the HIM (not to scale) near the 3′ DRJ. (B) Magnification of the 121-bp HIM, showing two regions with many chimeric reads, called J2 (left) and J1 (right). (C) Sequence logo of J2 and J1 generated with weblogo.berkeley.edu, using an alignment of the HIMs of the 16 segments that integrated into the S. nonagrioides genome. For J2, we used the 30 bp upstream from the minimum position at which we observed >2 chimeric reads; for J1, we used the 30 bp downstream from the maximum position at which we observed >2 chimeric reads. The highly conserved motif J1 is framed in red and J2 in green.

To identify and quantify integrations of C. typhae bracovirus (CtBV) DNA circles into the S. nonagrioides genome, we searched for chimeric reads for which a region aligns on a CtBV proviral segment and the other region aligns on the caterpillar genome. We identified chimeric reads mapping to all 16 C. typhae proviral segments containing a HIM in all 5 DNA samples. The total number of chimeric reads (excluding PCR duplicates) on these segments varied from 4 on segment 16 in the head to 947 on segment 1 in the hemolymph. Importantly, between 87.5% and 100% of chimeric reads mapping to the 16 HIM-containing segments were located in HIMs (see Fig. S3). In fact, the vast majority of chimeric reads mapped to two short regions located within HIMs and spaced by 41 to 73 bp (see an example of segment 1 in Fig. 3; also see Fig. S3). Alignment of all 16 HIMs allowed us to identify a conserved motif under each of these regions that corresponded to the J1 and J2 junctions previously characterized in C. congregata and M. demolitor (Fig. 3C) (26, 30). Overall, the pattern we observed confirms that HIMs split during circle integration, that the 41- to 73-bp region between J1 and J2 is lost, and that J1 and J2 end up at the extremities of the linearized circle once in the host. Our results also show that the 16 HIM-containing circles integrate in all host tissues surveyed and that most integrations of these circles into the S. nonagrioides genome are mediated by HIMs.

### Potential role of microhomologies in CtBV circle integration.

Interestingly, wasp-moth junctions in chimeric reads do not all map at the same position within the J1 or J2 motifs. Rather, they are distributed over 2- to 12 bp-long regions, depending on the segments and samples (Fig. 3; also see Fig. S3). This pattern could be due to biological variation in the position of the breakpoint within the J1 and J2 motifs. It could also reflect imprecision in our mapping of wasp-host junctions caused by the presence of microhomologies between CtBV and host sequences at the junction. Indeed, at the CtBV-host junction, there is a 1 in 4 chance that the base following the junction position in the CtBV circle would be the same as that following the junction in the moth genome (see Fig. S4a). In our approach, the position of the CtBV-host junction corresponds to that of the BLASTN alignment end coordinate on the wasp, regardless of the presence of any overlap (see Fig. S4b). There is thus a 1 in 4 chance for the true position of the CtBV-host junction to be shifted by 1 bp for an overlap of 1 bp. For an overlap of 2 bp, there is a 1 in 16 chance that the wasp-host junction would be shifted by 2 bp. Interestingly, when there is no overlap, we observed that the CtBV-host junction almost always occurs at the same exact position in J1 and J2 for all segments, with some very rare chimeric reads shifted by 1 bp. Thus, it appears that junctions devoid of microhomology involve CtBV circles that all underwent a double-strand break at the same exact position, as expected under the hypothesis that bracovirus circle integration is mediated by a site-specific recombinase (30). Among junctions with microhomology, we found more chimeric reads with shifted CtBV-host junctions than expected by chance, suggesting that the imprecision of the breakpoint may be at least partly biological.

**FIG 4:**
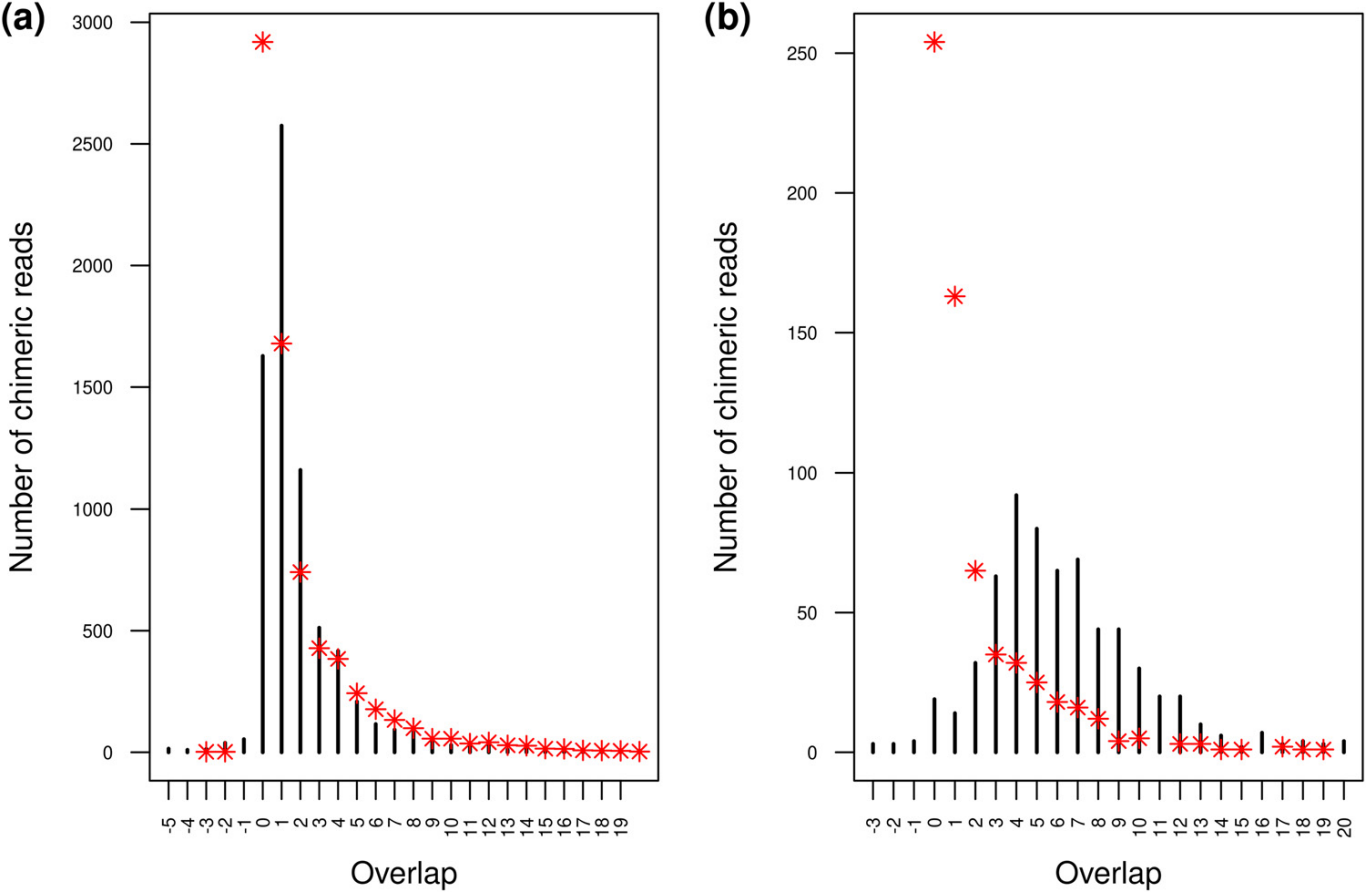
Distribution of microhomology lengths at wasp-host junctions in chimeric reads. Black bars correspond to the numbers of observed chimeric reads for each microhomology length. Red asterisks correspond to the expected numbers of chimeric reads for each microhomology length. (a) Distribution of microhomology lengths for CtBV-host junctions mapped in J1 or J2. (b) Distribution of microhomology lengths for CtBV-host junctions mapped within HIM but outside J1 or J2.

To further assess whether bracovirus-moth microhomologies at the junctions may somehow foster integration of DNA circles, we compared the expected numbers of chimeric reads for each microhomology length to the observed values (see Materials and Methods) (Fig. 4). We did this for chimeric reads falling specifically in J1 or J2 and for chimeric reads falling outside J1 and J2 but still in the HIM regions. Regarding chimeric reads falling in J1 or J2, we found that the number of observed microhomology lengths was close to that expected by chance for microhomology lengths of >3 bp. Thus, while certainly affecting the precision of our junction-mapping pipeline, these microhomologies are unlikely to have biological underpinnings. In contrast, the numbers of 0-bp, 1-bp, and 2-bp microhomologies differed markedly from what is expected by chance, with the observed 0-bp microhomologies being 1.8 times less numerous and 1-bp and 2-bp microhomologies being 1.5 times more numerous than expected by chance (Fig. 4a). Like 3-bp-long microhomologies, 1-bp- and 2-bp-long microhomologies affect the precision of our junction-mapping pipeline. However, their overrepresentation indicates that they likely have biological underpinnings. For chimeric reads falling outside J1 and J2 but still in the HIMs, we observed a major underrepresentation of 0- to 2-bp microhomologies (65 versus 478 reads), mirroring a major overrepresentation of 4- to 13-bp microhomologies (478 versus 105 reads). This suggests that, when the breakpoint is located further away from the canonical positions of J1 and J2, the presence of microhomology between CtBV and moth sequences may be crucial for successful integration.

### Few integrations of CtBV DNA circles outside HIMs.

Our search for chimeric reads also yielded a number of reads mapping outside HIMs in HIM-containing segments, as well as reads mapping to segments that did not contain a HIM. The number of such reads was low. In HIM-containing segments, the number varied from 0 (for 11 segments in all or some tissues, depending on the segment) to 23 (for segment 1 in the hemolymph). In segments devoid of HIM, this number varied from 1 (for multiple segments in multiple tissues) to 11 (for segment 20/33 in the fat body). In contrast to chimeric reads mapping to HIMs, which are clustered in two regions corresponding to J1 and J2 motifs, reads mapping outside HIMs are dispersed over the circles, with no circle position outside HIMs being mapped by more than one bracovirus-host junction, except for two junctions covered by 2 reads each. This pattern could suggest that, in addition to HIM-mediated integration, circles could integrate into the S. nonagrioides genome through other mechanisms, possibly involving host DNA repair pathways, as suggested by Wang et al. (31) for Diadegma semiclausum ichnovirus (DsIV). In agreement with this, we found that the number of chimeras mapping outside HIMs in bracovirus circles was always higher than expected, given the number of wasp-host chimeras involving exons of wasp BUSCO genes (see Materials and Methods). However, given the small number of such non-HIM, bracovirus circle integrations, this possible alternative circle integration mechanism is unlikely to play a significant role in parasitism for C. typhae.

### Gene content of integrated segments.

**FIG 5:**
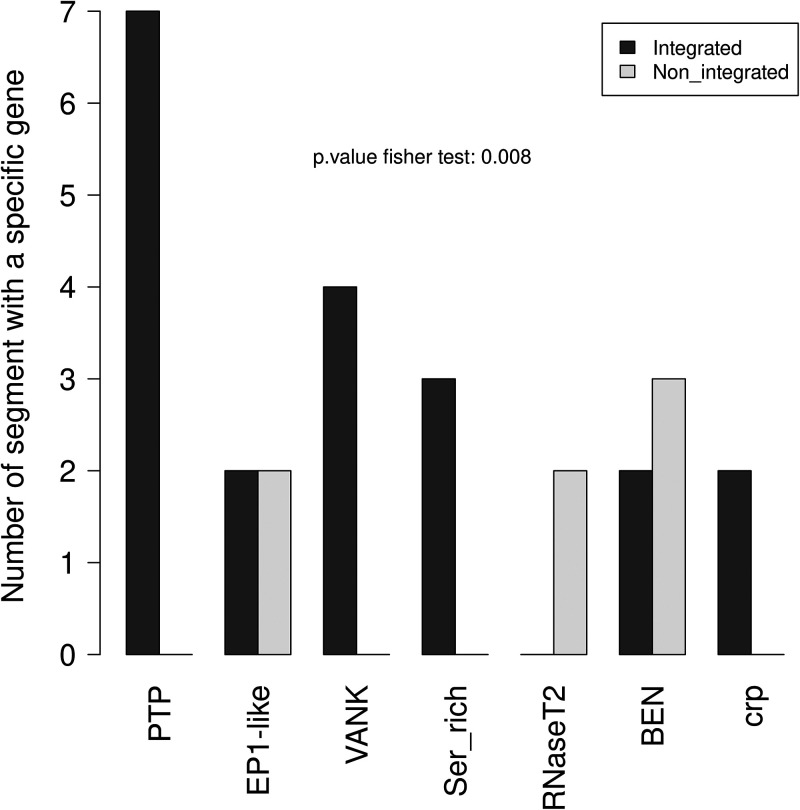
Integration capacity of segments containing ≥1 gene belonging to seven gene families: PTP (protein tyrosine phosphatase), EP1-like (early parasitism-specific protein 1), VANK (viral ankyrin), Ser_rich, RNaseT2, BEN (BEN-domain proteins), and crp (cysteine-rich proteins). Segments containing genes belonging to several gene families are counted for each family. Black bars correspond to segments that integrate into the genome of S. nonagrioides, while white bars correspond to segments that do not integrate.

To assess whether circle integration is associated with circle gene content, we compared gene family content for integrated versus nonintegrated circles (Fig. 5). This comparison was done for all gene families with known predicted domains and more than 2 genes. Overall, it appears that the integration of a circle is associated with its content in gene families (Fisher exact test, *P* < 0.01). Three gene families present on at least 3 segments seem to explain this observation, i.e., viral ankyrin (VANK), serine-rich, and protein tyrosine phosphatase (PTP). These gene families contain 5, 4, and 24 genes distributed over 4, 3, and 7 segments, respectively, all found integrated in the S. nonagrioides genome. This observation suggests that integration of these three gene families is important for parasitism success.

### Quantification of integrated bracovirus circles in the S. nonagrioides genome.

**FIG 6:**
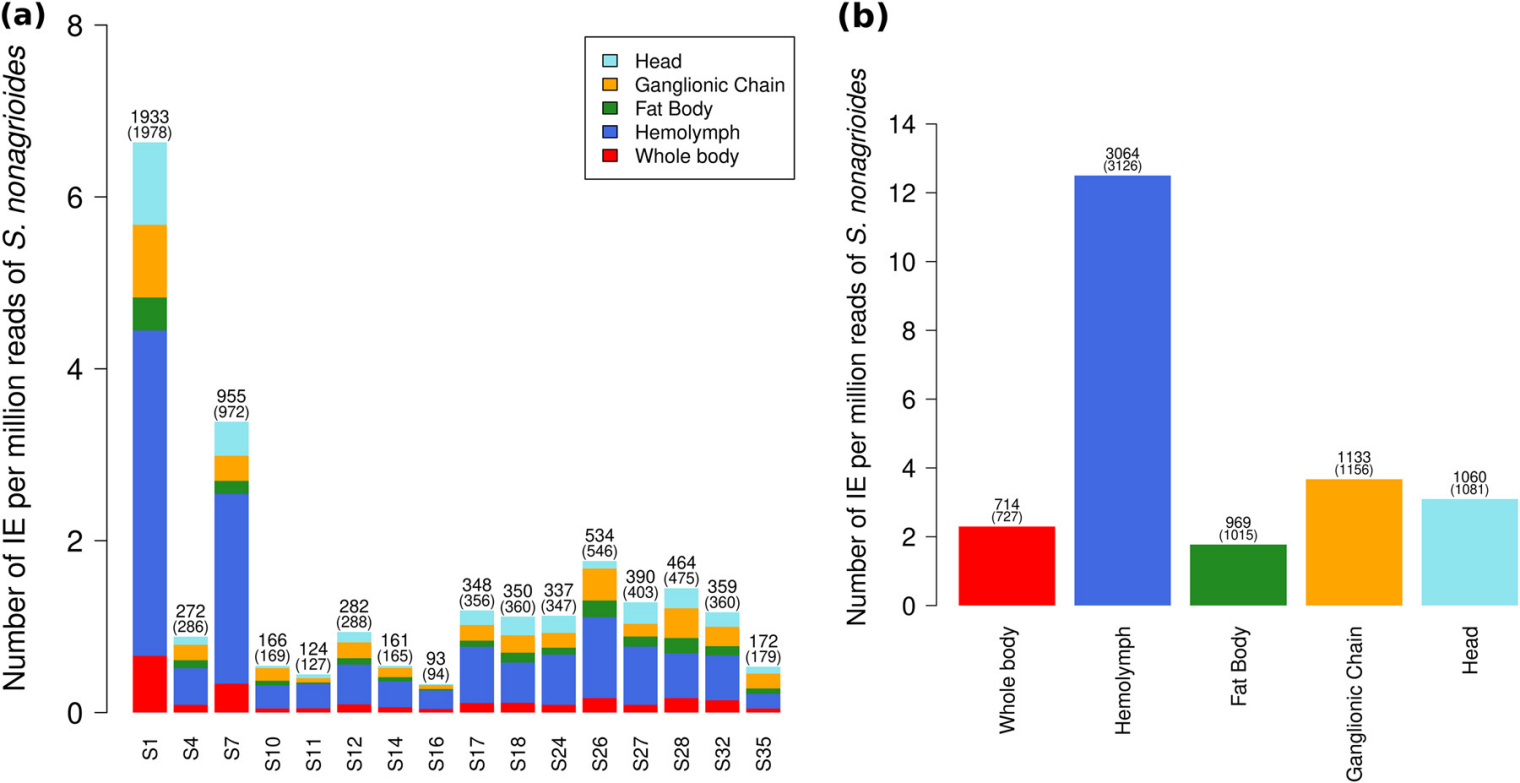
Number of IEs for each segment and sample. Absolute numbers of IEs and of chimeric reads (in parentheses) are shown at the top of each bar. (a) Barplot comparing the numbers of IEs for each segment. (b) Barplot comparing the total numbers of IEs of all segments in each sample.

We then set out to quantify the number of integrations of CtBV circles that occurred during parasitism of S. nonagrioides larvae in our experiment. Parsimoniously, we considered only chimeric reads that fell in the J1 and J2 motifs of the HIMs, that is, between 730 and 3,126 chimeric reads per sample. We found that the vast majority of integrations in the moth’s genome (6,784 [98%] of 6,940 integration events [IEs]) were supported by 1 chimeric read only. Among IEs supported by more than 1 read (2%), 3 were supported by 3 chimeric reads and the rest by 2 chimeric reads. This pattern indicates that most chimeric reads correspond to independent IEs. Thus, among the host cells we sequenced, almost no cells harbored a shared IE that would originate from a cell division. Figure 6 shows the number of IEs we inferred per segment and per sample by counting each integration position only once. This led to 714 to 3,064 IEs, depending on the samples and segments. In order to be able to compare the number of IEs for each segment between samples, we turned absolute numbers of IEs into relative numbers normalized to 1 million reads mapping to the genome of S. nonagrioides (see Table S6). Considering all samples together, S1 is the segment with the most integrations, with 6.64 IEs per million reads mapping to the host (S. nonagrioides) (IPMH), followed by S7, with 3.38 IPMH and then by S26 with 1.76 IPMH (Fig. 6a). All of the other segments have less than 1.45 IPMH. For all segments, the hemocyte sample is the sample with the most chimeric reads (Fig. 6a). In total, we infer about 12.5 IPMH in the hemocytes, about 3 IPMH in the ganglionic chain and the head, and about 2 in the whole body and the fat body (Fig. 6b). Given the haploid genome size of S. nonagrioides (1,021 Mpb [40]), the read size (150 bp) and the number of IPMH, we estimate the average number of IEs per genome as follows: (IPMH/read size) × genome size (in mega-base pairs). This yielded an average of 85, 25, and 12 IEs per genome in the hemocytes, ganglionic chain, and fat body, respectively.

### Quantification of HIM-containing CtBV circles in their integrated versus circularized forms.

**FIG 7:**
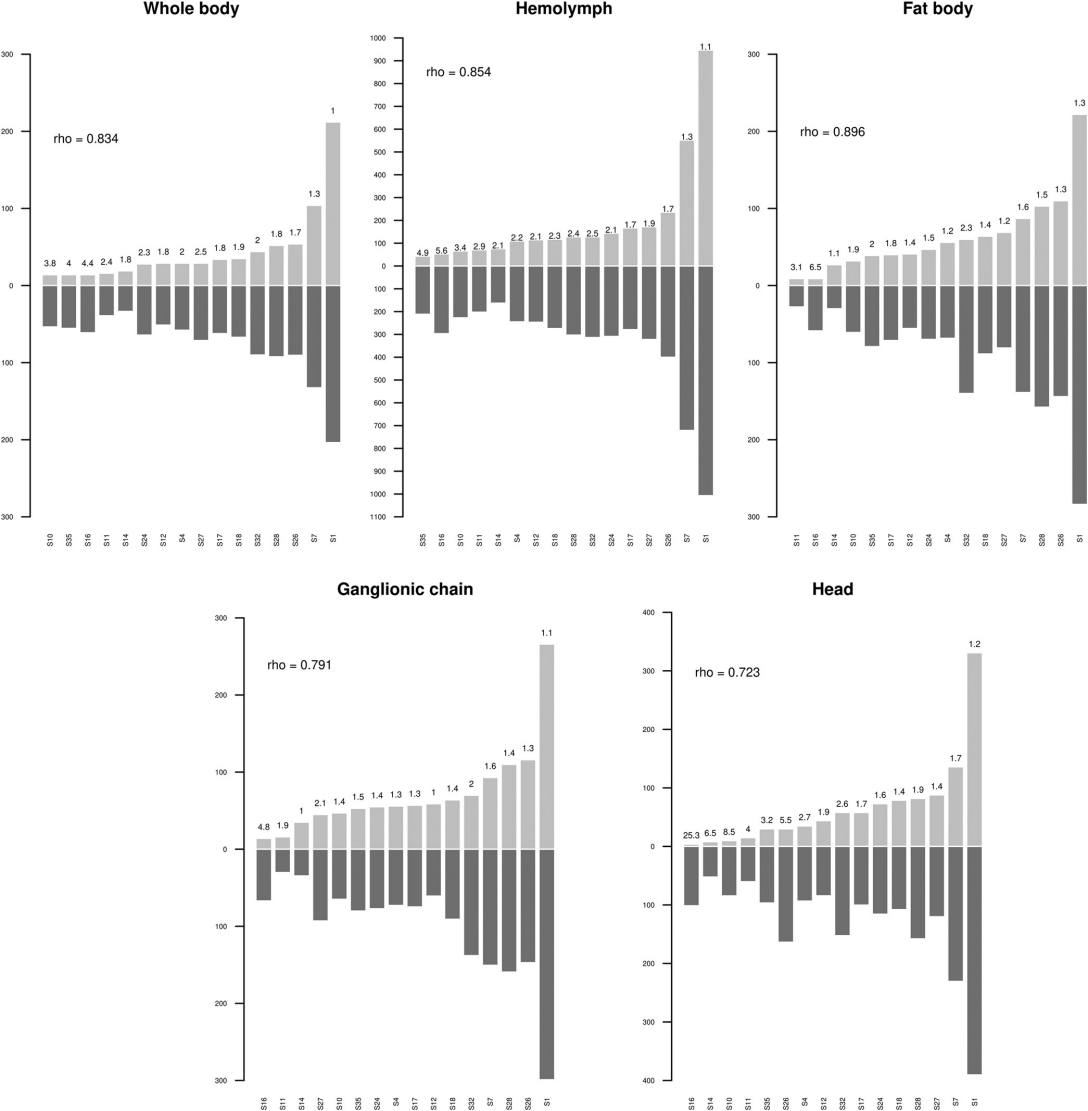
Histograms showing the number of chimeric reads and the sequencing depth for each HIM-containing segment. Light gray bars show the number of chimeric reads, while dark gray bars show the sequencing depth. The ratio of sequencing depth to chimeric reads is indicated at the top of each light gray bar. The Spearman rho values indicate the correlation between sequencing depth and the number of chimeric reads for each sample.

We assessed how many of the HIM-containing CtBV circles we sequenced were integrated into the S. nonagrioides genome versus how many there were in total, regardless of their form (circular or integrated). For this, we compared the numbers of IEs (as an approximation of the number of sequenced integrated circles) to the average circle sequencing depths (as an approximation of the total quantity of CtBV) for each circle in each sample. We found that the numbers of IEs per circle were strongly correlated with sequencing depths for all samples (Spearman rho of 0.7 to 0.9; *P* < 0.01) (Fig. 7). This indicates that the number of integrated circles depends to a relatively large extent on the total amount of circles that are injected by wasps into their host. Interestingly, we also found that the ratio of any forms to integrated circles varied depending on the circles, with these variations being similar among samples. For example, circle 1 is characterized by the lowest ratio in 3 of 5 samples, while circle 16 has the highest ratio in all samples (Fig. 7). This likely reflects variation in the efficiency of integration among circles. In addition, it suggests that a significant part of the circles remain nonintegrated, at least for the circles with a high ratio. Thus, in addition to being determined by the overall quantity of circles injected by the wasp, the propensity of a circle to integrate depends on other factors, possibly the binding affinity of the integrase/recombinase to HIM sequences, which may have more or less diverged from the optimal HIM.

### Persistence of nonintegrated circles 7 days postoviposition.

**FIG 8:**
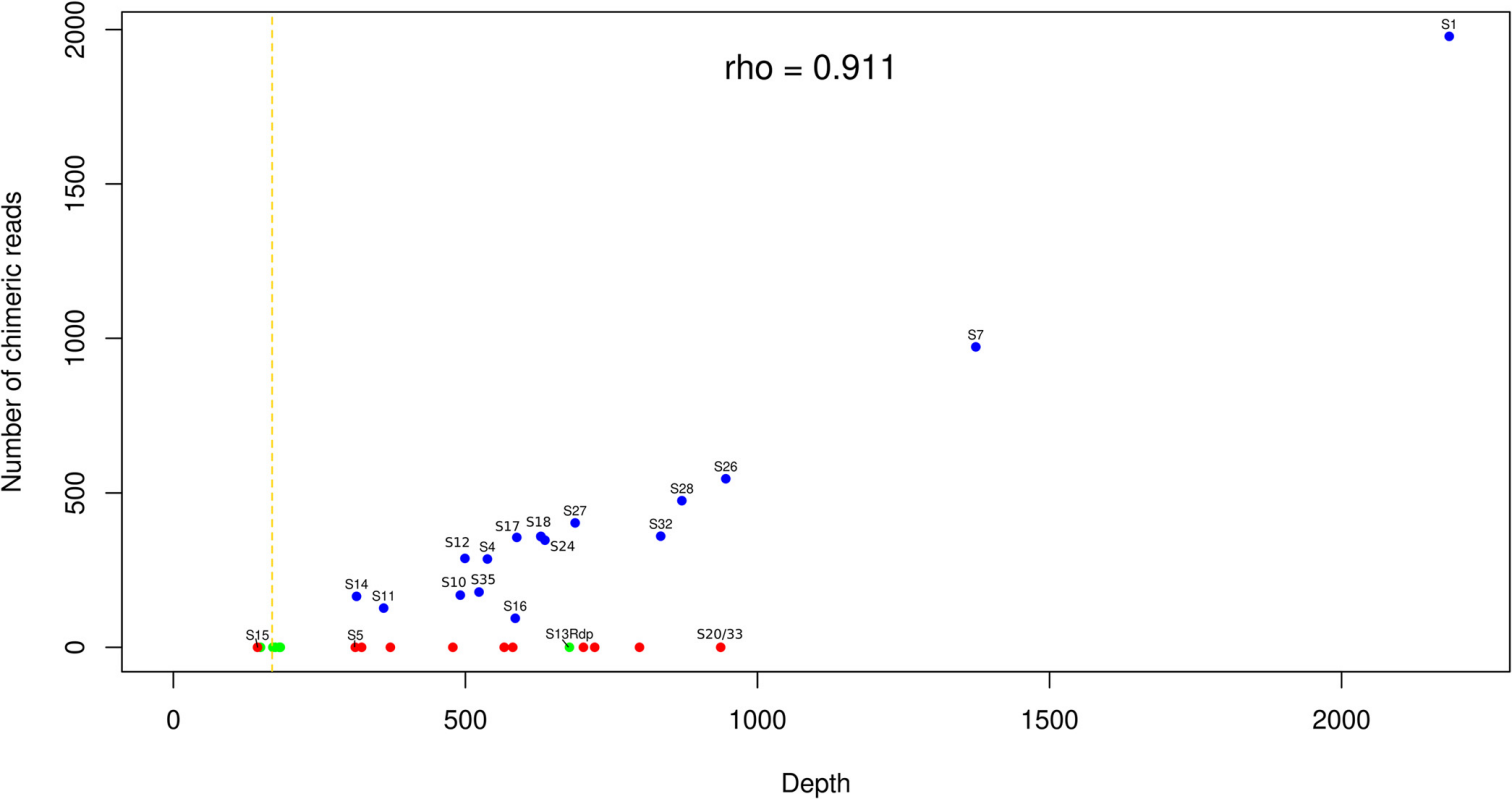
Plot of the sequencing depth versus the number of chimeric reads for each of the CtBV segments. Sequencing depths and numbers of chimeric reads were summed for all samples. The same plots are shown for each sample in Fig. S5 in the supplemental material. Blue dots represent proviral segments that do integrate into the S. nonagrioides genome, and red dots represent proviral segments that do not integrate. Green dots represent duplicated segments, i.e., Rdp and Hdp segments. The identification numbers of the segments are shown near each blue dot. For red dots, only the identification numbers S15, S5, and S20/33 are shown; for green dots, only S13_Rdp is indicated. The yellow dashed line shows the average depth on the C. typhae genome when all samples are summed. The Spearman rho value indicates the correlation between sequencing depth and the number of IEs for segments that do integrate into the S. nonagrioides genome.

We then used the average sequencing depth per circle to compare the quantity of HIM-containing circles (or circles that integrate into host genomes) to the quantity of other circles, which do not integrate into host genomes (Fig. 8; also see Fig. S5). The sequencing depth of segment 15 is close to the average depth on the C. typhae genome, suggesting that this segment is present only in the form of proviral segments in C. typhae cells that are present at different levels in the different tissues. This is in accordance with the annotation of this segment, for which we did not find any DRJs, suggesting that segment 15 is not able to form DNA circles and should thus be considered a pseudosegment (Ps15 in Fig. 1). All other segments display higher coverage than the C. typhae genome, suggesting that, in addition to their proviral form present in C. typhae cells, they are present in the circular form and/or in the integrated form. We found that, with the exception of circles 1 and 7, which are characterized by very high sequencing depths and large numbers of IEs, the ranges of sequencing depths were similar between HIM-containing circles (from 314 to 946) and other circles (from 311 to 937, leaving Ps15 aside) (Fig. 8; also see Fig. S5). Thus, the number of integrating circles found in host tissues 7 days postparasitism is similar to that of nonintegrating circles.

In addition, it is worth noting that the sequencing depth of S13_Rdp in the parasitized caterpillars was in the range of that of the functional segments (Fig. 8). This observation supports the presence of the parental S13 in C. typhae, which we were not able to assemble. The high sequencing depth of S13_Rdp is likely due to the fact that reads that would map onto S13 if it were in our assembly instead map to the very similar S13_Rdp. Importantly, the sequencing depths of all other Rdp and Hdp segments were in the range of the sequencing depths of the other regions of the C. typhae genome, supporting the idea that they are present only as proviral segments in teratocytes and other residual wasp cells. This is in line with the nonfunctional nature of these duplicated segments, which are not expected to generate circles (Fig. 8).

### Distribution of wasp segments throughout the genome of S. nonagrioides.

We investigated whether DNA circles integrate randomly along the S. nonagrioides genome. To do so, we split the genome into 100,000-bp windows and assessed whether some windows were subjected to more integrations than expected by chance. We chose to not have any windows with a mixture of contigs, which led to 2,553 windows smaller than 100,000 bp that we eliminated from our analysis. The remaining 9,121 windows of 100,000 bp covered 89.3% of the genome and bore 88.3% to 90.7% of the IEs, depending on the samples. Under the null hypothesis that segments integrate at random in the S. nonagrioides genome, we assumed that the number of windows bearing integrations followed a Poisson distribution. We compared observed versus expected numbers of IEs under our null hypothesis for each sample separately as well as for a pool of all samples. Observed distributions departed significantly from the expected ones in all 6 cases (*P* < 0.001) (see Fig. S6). In the case of the pooled data set, we observed an excess of windows with no IEs or ≥3 IEs and we observed a deficit of windows with 1 or 2 IEs. Under a Poisson distribution, we did not expect any windows with more than 7 IEs. However, some windows have up to 23 IEs. This result suggests that segments do not integrate entirely randomly into the genome, as observed for C. congregata bracovirus (CcBV) in the genome of M. sexta (30). Interestingly, two windows had ≥2 IEs in all 5 samples, with a maximum of 8 IEs. These IEs come from 9 and 11 segments; therefore, the overrepresentation of IEs in these two windows is not due to 1 specific segment targeting them. We then tested whether several factors, including variation of sequencing depth, GC content, TE content, or gene content, could explain the distribution of IEs along the S. nonagrioides genome. We found that none of these variables was strongly correlated with the IE density (data not shown).

## DISCUSSION

### HIM-mediated duplications of CtBV in the wasp genome.

In this study, we assembled a high-quality genome for the braconid wasp C. typhae, which allowed us to annotate 27 bracovirus proviral segments plus 7 other duplicated segments, among which at least 6 resulted from HIM-mediated duplications. Such a high number of HIM-mediated duplications is noteworthy, given that none was found in the high-quality assembly of C. congregata. These duplications imply that, after circularization, a segment can integrate into the genome of wasp germ line cells, as suggested by Serbielle et al. (39), who identified 3 HIM-mediated duplications in C. sesamiae. Several scenarios could explain such accidental integration. First, HIM-mediated duplication in the wasp could occur through ectopic circularization of a segment in germ cells associated with ectopic expression of wasp factors required for integration in these cells. This scenario appears unlikely, because it would imply that the entire set of complex processes leading to particle production would accidentally occur in the germ line. Second, circle-containing bracoviral particles could sometimes accidentally enter germ cells of the wasp that produced them. This scenario also seems unlikely, because virus particles are released in the calyx lumen, which is located in the posterior part of the ovaries, whereas germ line cells are located in the upper part, in the ovarioles. Third, circle-containing bracoviral particles could enter another wasp individual during accidental oviposition in this individual. This could occur when a wasp oviposits in an already parasitized host larva containing a high density of wasp embryos, as observed for Microplitis croceipes (41). Such behavior could occur more frequently in wasps parasitizing stem borer hosts, because these wasps follow the galleries made in the plant stem by their host, instead of ovipositing from outside the plant (42). The aggressive response of stem borer hosts may indeed impose a higher pressure on female wasps in the confined, plant stem environment, which may be more conducive to behaviors such as oviposition into host larvae that have already been parasitized by another wasp. In this respect, it is noteworthy that we found HIM-mediated duplications only in *Cotesia* wasps parasitizing stem borers (C. typhae, C. sesamiae, and C. flavipes). No such duplications were found in the three other *Cotesia* species (C. rubecula, C. glomerata, and C. congregata), which are known to parasitize lepidopteran hosts dwelling on plant leaves (43). Whether the type of host and its habitat have an impact on the likelihood of HIM-mediated duplications will have to be reappraised when higher-quality genomes are available for other wasps.

Several clues suggest that HIM-mediated duplications may participate in the dynamic evolution of wasp bracoviral segments. Indeed, there are striking similarities in the gene content between segments producing DNA circles in the PL2 region and isolated loci (such as S1 in PL2 and S17 in PL3) in *Cotesia* species, which suggests that dispersed loci may originate from duplications, whether these duplications are mediated by HIM or not (39). However, the generation of a new segment by HIM-mediated duplication is probably rare, since such a segment would need complex genome rearrangements in order to acquire the ability to form DNA circles. Indeed, after HIM-mediated duplication, the segment contains a single DRJ (whereas both the 5′ DRJ and the 3′ DRJ are required for circularization) and none of the regulatory sequences allowing bracovirus DNA amplification, which are located at the extremities of the amplified regions (RUs) outside proviral segments (3, 21). Duplications could also participate in the dynamic evolution of wasp bracoviral segments through gene conversion or other mechanisms.

### Chromosomal integration of CtBV in multiple host tissues.

This study shows that all 16 HIM-containing circles of CtBV undergo chromosomal integration in S. nonagrioides cells during parasitism. Previous studies characterizing chromosomal integration of polydnavirus DNA circles focused only on one tissue type (hemocytes) and/or were limited in terms of the number of circles studied (26, 28–31). Here, we used bulk Illumina sequencing of DNA extracted from hemocytes, fat bodies, ganglionic chains, and heads of parasitized S. nonagrioides larvae, which shows that chromosomal integration of DNA circles is not limited to hemocytes and extends to all other surveyed tissues. Interestingly, the C. typhae genome has been sequenced deeply (110×) in the hemolymph, which indicates the presence of numerous wasp cells in this tissue. These wasp cells may be teratocytes, which are known to be released from the wasp embryonic membranes into the host when eggs hatch. In C. congregata, which is gregarious at the larval stage, like C. typhae, the number of teratocytes reaches about 140 teratocytes per wasp embryo (44). Teratocytes play various roles during parasitism, including host immunosuppression, production of antimicrobial peptides, and nutritional functions (45, 46). They undergo physiological and morphological changes during development of the wasp embryos, including an increase in ploidy level (47, 48), which likely explains the high sequencing depth obtained over the C. typhae genome in the hemolymph. In the other tissues, however, the sequencing depth for C. typhae was much lower (down to 3× in the fat body), indicating that few if any wasp teratocytes were sequenced in those samples. This in turn points to a low level of contamination of other tissues by hemolymph, indicating that the majority of CtBV circle IEs we identified in nonhemolymph tissues are *bona fide* chromosomal integrations in cells constituting those tissues. Although we did not perform replicates for each tissue, our study seemingly indicates that the number of circle IEs is higher in hemocytes than in other tissues, which is in line with the role of hemocytes in immunity (49, 50) and the known effect of circle-borne virulence genes in thwarting host immunity responses (2). This could also indicate that hemocytes are preferentially infected by CtBV, resulting in greater abundance of viral circles in these cells, as already suggested by Beck et al. (51). Given the multiple effects of bracovirus on host physiology, development, and behavior (29), it is likely that integration in a wide range of tissues contributes to parasitism success and is not merely a by-product of the capacity of bracoviral particles to enter many cell types.

### Persistence of integrated versus nonintegrated CtBV circles.

It was traditionally assumed that integration of polydnavirus circles was beneficial to the wasp because it allowed persistence and expression of these circles throughout the duration of wasp embryonic and larval development, which can last between 7 and 14 days under laboratory conditions, depending on the species considered (26, 30, 52). Here, we estimated that, at 7 days postoviposition, parasitized hosts contain between 12 and 85 integrated circles per haploid genome, depending on the tissue. Most IEs characterized in this study are supported by only 1 chimeric read, indicating that most integrations are specific to one of the S. nonagrioides cells we sequenced. At first sight, this may seem unexpected, because an IE occurring early after parasitism may be expected to be shared by many cells at 7 days postparasitism as a result of successive divisions of the original IE-bearing cell. However, given the range of haploid genome sequencing depths (70× to 155×, depending on the samples), we estimated that we sequenced a very small number of S. nonagrioides cells (maximum of 35 to 77 cells). Thus, the probability of sequencing 2 cells with the same IE was very low. Therefore, the fact that we find very few IEs supported by more than 1 chimeric read cannot be taken as an indication of limited persistence of integrated circles in a given host cell lineage through successive mitotic divisions. Measuring such persistence would require sequencing the host genome more deeply. However, an interesting observation we made regarding the persistence of circles throughout parasitism is that, with the exception of circles 1 and 7, the 14 other integrating circles are not present in greater quantities than nonintegrating circles, a trend that holds for all tissues (Fig. 8; also see Fig. S5 in the supplemental material). Thus, it appears that similar quantities of integrating and nonintegrating circles can persist over at least 7 days during parasitism. It follows that integration is not a requirement for persistence during at least one-half of the duration of C. typhae embryonic development. Interestingly, our data confirm that integrating and nonintegrating circles clearly differ in terms of gene content, with genes such as VANK and PTP being present exclusively on integrating circles (Fig. 5) (30). Further studies are needed to shed light on the role of integration during parasitism (30) and on the link between CtBV gene content and integration.

### Mechanism of CtBV integration.

Our study confirms that bracovirus DNA circles integrate into the genome of wasp hosts through site-specific recombination involving HIMs (26, 30). As proposed earlier, *vlf-1* and *int-1*, two candidate genes of nudiviral origin encoding an integrase domain (of the phage integrase family also known as tyrosine recombinases) may be involved in chromosomal integration (30). These two proteins are loaded into bracovirus particles (16, 53) and delivered to the host, and they were shown by RNA interference experiments to be involved in circle excision (54). Interestingly, our study of microhomologies between wasp and moth sequences at bracovirus-moth junctions reveals variation in the mechanism of integration. On one hand, we found that all bracovirus-moth junctions devoid of microhomology, which represent 21% of all junctions uncovered in this study, occur at the exact same positions in the J1 and J2 motifs within the HIM. This may indicate that, for a relatively large fraction of integrations, the occurrence of a double-strand break at a canonical position can be resolved without microhomology. On the other hand, we found an excess of 1-bp and 2-bp microhomologies in the junctions located in J1 and J2 motifs and an excess of 3-bp to 12-bp microhomologies in the junctions located outside J1 and J2 motifs (641 [8%] of 7,746 reads). Thus, our results indicate that integration can occur with or without wasp-moth base pairing, but it is unclear whether microhomology-mediated integrations are generated through the same mechanism as integrations devoid of microhomology (30) or whether they may occur through DNA repair mechanisms (55).

### Evolution of HIM in braconid and ichneumonid wasps.

Irrespective of whether chromosomal integration of wasp circles involves a single mechanism or multiple mechanisms, it appears that the vast majority of IEs, if not all of them, occur at double-strand breaks generated within HIMs. Our study thus confirms the central role played by HIMs in integration. While these motifs have been found in all Microgastrinae wasps studies so far, they could not be identified in *Chelonus inanitus*, a bracovirus-containing microgastroid wasp belonging to another subfamily (Cheloninae) (30). It was thus proposed that HIMs might have been acquired by the ancestor of Microgastrinae about 54 million years ago, independently and well after the domestication of the nudivirus shared by all microgastroid wasps (30). The recent finding that ichnovirus circles from the ichneumonid wasp *Diadegma semiclausum* undergo chromosomal integration into their host via HIM-like motifs raises the question of the evolutionary link between these motifs in bracoviruses and ichnoviruses. Structurally, ichnovirus and bracovirus HIMs are similarly made of J1 and J2 motifs separated by a stretch of sequence that is deleted upon circle integration. The size of the sequence between J1 and J2 is relatively homogeneous in most bracovirus and ichnovirus segments (33 to 78 bp), although some ichnovirus segments have longer intervening sequences (e.g., DsIV-38 and Tranosema rostrale ichnovirus F1, which have 311-bp-long and 1,781-bp-long intervening sequences, respectively). Like bracovirus HIMs, which do not seem to be ubiquitous among microgastroids (i.e., they were not found in *Chelonus inanitus* bracovirus segments), ichnovirus HIMs were not found in all Campopleginae wasps known to harbor an ichnovirus that were searched (31). Indeed, in addition to DsIV, Wang et al. found HIMs in *Tranosema rostrale* and *Hyposoter fugitivus* ichnoviruses but not in *Campoletis sonorensis* ichnovirus (31). We think that three evolutionary scenarios could explain the presence of HIMs in both bracovirus and ichnovirus segments. The first scenario posits that HIMs and other integration factors were present in the ancestor viruses (bracovirus and ichnovirus) and were lost in several wasp lineages. Supporting this scenario, nudivirus HzNV1 is known to integrate into the DNA of cultured cells and to persist during a latent phase both as an integrated form and as an episomal form (56). Nudivirus integration properties might have favored the recurrent domestication of nudiviruses by parasitic wasps (9, 11). However, the mechanism of HzNV1 integration has not yet been characterized. Concerning ichnoviruses, because the ancestor belongs to a virus family that is possibly extinct, nothing is known regarding potential ancient integration properties. The second scenario assumes that integration of DNA circles evolved after viral domestication. It implies that HIMs would have been acquired in Braconidae and Ichneumonidae after viral domestication. This acquisition could have occurred through independent recruitment of recombinase sites and proteins from related viral elements or TEs present in both braconid and ichneumonid wasp genomes. In agreement with this scenario, HIM-like motifs that contain inverted terminal repeats and are involved in site-specific recombination are common in prokaryotes, yeast, and viral genomes and TEs (57). Recombination sites of site-specific recombinases involved in DNA insertions, inversions, or circularizations are typically between 30 and 200 nucleotides in length and consist of two motifs with a partial inverted repeat symmetry, to which the recombinase binds and which flank a central crossover sequence at which the recombination takes place (58). HIM sites correspond fairly well to that description. In eukaryotes, several examples of recombinases originating from TEs have been reported, such as the RAG1 protein, which is responsible for shuffling immunoglobulin genes in vertebrates (19), and transposases that are involved in the maturation of paramecium nuclei (59). Finally, a third scenario would imply that HIMs were acquired only once, by either Ichneumonidae or Braconidae wasps, and then were transferred between the two polydnaviruses. Such transfer could have been favored by the integration properties of polydnavirus circles and by the fact that some wasps from the two families are known to parasitize the same host species (60, 61). This seems rather unlikely, however, since it is not sufficient to transfer the HIM sequence to provide a functional mechanism, and the recombinase gene needs to be transferred at the same time; however, the latter is not present on a bracovirus circle (it is packaged as a protein in polydnavirus particles). Characterization of the different proteins involved in circle integration and of the integrases/recombinases encoded in parasitoid wasp genomes will probably be helpful to shed further light on the evolutionary history of HIMs and polydnaviruses at large.

### Possible long-term impact of polydnavirus integration in wasp hosts.

Previous studies uncovered bracovirus circle sequences in the genomes of several species of lepidopterans, indicating that such sequences were horizontally transferred from wasps to lepidopterans at some point during the evolutionary history of these insects (18, 62). Although we did not include host germ line tissues in this study, our finding that bracovirus circles can integrate into host tissues other than hemocytes suggests they may also integrate into host germ line cells. In this context, it is remarkable that a fairly large number of bracovirus integrations were found in the whole-body sample (Fig. 6), i.e., a host larva in which no wasp larvae were present 7 days postparasitism. The absence of wasp embryos in this larva and the 5 larvae that we did not sequence could be due either to active resistance of the host, which would have prevented the development of these embryos, or to the fact that the wasp injected venom but no eggs into these larvae (63). Relatively high sequencing depths over the entire C. typhae genome in the sequenced larva (Fig. 2) are in agreement with a possible presence of teratocytes, in turn suggesting that eggs were indeed injected by the wasp. Although we could not assess whether the sequenced larva would have developed into an adult and been fertile, we have verified by PCR the presence of bracovirus circles in several adults of S. nonagrioides that survived parasitism by C. typhae in our laboratory (data not shown). Altogether, these results tend to support the hypothesis according to which wasp-to-lepidopteran horizontal transfer of bracovirus segments can occur through HIM-mediated integration.

## MATERIALS AND METHODS

### DNA extraction, library preparation, and sequencing of the C. typhae genome.

The DNA extraction was performed on C. typhae individuals from an isofemale line that has been reared in the Evolution, Génomes, Comportement, et Écologie (EGCE) laboratory (Gif-sur-Yvette, France) since 2015, from a strain reared at the International Centre of Insect Physiology and Ecology (Nairobi, Kenya) since 2013, when it was initially collected from the Kobodo locality in Kenya (0.679S, 34.412E). In order to obtain high-quality DNA, several individuals were pooled and ground in liquid nitrogen to give 100 mg of fine dry powder. The DNA was then extracted using Nucleobond AXG100 columns and buffer set IV from Macherey-Nagel, following the manufacturer's protocol. We obtained 26 μg of DNA, quantified with a Qubit fluorometer (Thermo Fisher Scientific). The integrity of DNA was checked on an agarose gel, and Nanodrop measurements were performed to confirm the absence of proteins and other contaminants. For whole-C. typhae genome sequencing, we subcontracted the French National Sequencing Center (Genoscope, Evry, France) to prepare two types of DNA libraries according to the requirements for Illumina and ONT sequencing. The Illumina library was sequenced on a MiSeq platform using the 300-bp paired-end sequencing mode with a targeted mean insert size of 350-bp (see Table S1 in the supplemental material). Paired-end reads were trimmed of adapters and low-quality bases and then merged into single reads using the BBMerge tool (64). For Nanopore sequencing, preparation of libraries was carried out with a 1D genomic DNA ligation protocol (SQK-LSK109; ONT) and sequenced using R9.4.1 flow cells on both MinION and PromethION sequencers (ONT) (see Table S1).

### Assembly of the C. typhae genome.

The genome size was first estimated from a preliminary assembly obtained from Illumina reads with ABySS v2.0 (65) using a k-mer length of 96. The genome assembly was then performed *de novo* with Flye v2.5 (66) using 30× the longest ONT reads (see Table S1). The resulting Nanopore assembly was polished using Racon v1.5.7 (67) after mapping about 2 Gb of the longest raw ONT reads (see Table S1) with Minimap2 v2.17-r941 (68) and then Pilon v1.23 (69) using the merged Illumina reads mapped with BBMap v37.62 (70). The completeness of the genome assembly was assessed by searching for similarities to highly conserved genes among insects. For this purpose, we ran BUSCO v3.0.1 in genome mode, specifying a profile library of 1,658 single-copy core genes (April 2019 release) (35). Finally, scaffolds were checked for potential contamination by sequences from other organisms by visualizing them with Blobtools v1.1.1 using taxon-annotated GC-coverage plots. Blobtools assigns scaffolds to taxonomic ranks depending on their homologies, using both BLASTN (NCBI nucleotide database downloaded in November 2019) and BLASTX (UniRef90 protein database downloaded in November 2019). For each scaffold, Blobtools sums up scores of all hits by taxonomic rank and retains the best rank for the taxonomic assignment.

### Annotation of the C. typhae genome.

TEs were *de novo* identified and annotated in genomic sequences using TEdenovo and TEannot pipelines, respectively, included in the REPET package v2.5 (71). To construct a *de novo* repeat library, repeats were first screened using Recon (73), Grouper (72), and Piler (74). Consensus repeats were then classified into families using PASTEClassifier and filtered for all potential wasp genes corresponding to multigenic families. The TE library built by TEdenovo (71, 72) was then applied to perform a homology-based repeat search in the genome using TEannot (71, 72). Gene annotation was then performed on the repeat-masked assembly by running two iterations of MAKER v2.31.10 (75). The first iteration of MAKER used alignments of C. vestalis transcriptome assembly (est2genome = 1), and both reviewed Hexapoda and *Polydnaviridae* UniProt-Swiss-Prot proteins (January 2020 release) (protein2genome = 1) as sources of evidence for homology-based gene prediction. The resulting gene prediction was then used to train SNAP v2006-07-28 (77) and AUGUSTUS v3.3.2 (37) in order to construct *ab initio* gene models. The second run of MAKER allowed refinement of all of these gene models in a GFF3 output file. Predicted genes were functionally annotated with InterProScan v5.39-77.0 (76) using the PfamA database v32.0 (38) and with BLASTP v2.7.1+ using the UniProt-Swiss-Prot database (January 2020 release). Finally, functional annotations obtained were integrated in the final GFF3 file by using ipr_update_gff and maker_functional_gff modules distributed by MAKER.

### Annotation of CtBV proviral segments.

The localization of bracovirus proviral segments is relatively well conserved between species of the *Cotesia* genus and even with M. demolitor, which is more distantly related (3, 78). We annotated the proviral segments of C. typhae based on similarity searches using the proviral segments of its closest relative species (C. sesamiae and C. congregata) as queries. In *Cotesia congregata*, proviral segments are numbered from S1 to S37, including a segment that is no longer functional (pseudosegment 34 [ps34]) (20, 79). C. congregata has 36 proviral segments, and C. sesamiae has at least 26 proviral segments. The higher number of proviral segments in C. congregata results in part from extensions by duplications (responsible for 7 new segments at the macrolocus, for example) (20) and possibly from some losses in C. sesamiae.

The coding regions of the 26 segments of C. sesamiae (80) were aligned to the C. typhae genome using BLASTN to identify genes of each segment. DRJs of each C. congregata segment (see Data File S1 in the supplemental material) were then aligned using BLASTN searches for each homologous candidate segment in C. typhae to determine precisely the segment coordinates. The coding regions and DRJs of 10 segments present in C. congregata but not in C. sesamiae (segment S37new reported by Gauthier et al. [3], segments S3, S9, S19, S22, S29, and S31 in the macrolocus, and segments S10, S11, S21, and ps34 in dispersed loci [79]) were also aligned on the C. typhae genome. The synteny between segments and some other genes flanking the segments also helped to resolve ambiguous locations of the segments (3, 20).

### Annotation of HIM-mediated duplications of viral circle sequences in other *Cotesia* species.

We investigated whether any HIM-mediated duplications in C. typhae are shared with other *Cotesia* species, which would indicate that such duplications occurred before speciation. We used the chromosome-scale genome available for C. congregata and the more fragmented genomes of C. sesamiae, C. flavipes, C. rubecula, C. vestalis, and C. glomerata (3). In order to perform this analysis, we used the outputs of two BLASTN searches, (i) a similarity search between the *Cotesia* genomes and HIMs (HIMs of CtBV or CcBV, depending on whether the *Cotesia* species is more related to C. typhae or C. congregata) and (ii) a similarity search between the *Cotesia* genomes and the HIM-wasp genome junctions in C. typhae (options -max_target_seqs 5 -evalue 10e^−6^ for both searches). In the case of shared HIM-mediated duplications, we expect to obtain (i) hits on one-half of the HIM sequences for the first similarity search and (ii) hits on most of the length of the junctions for the second similarity search. Moreover, these two outputs should overlap; therefore, we filtered such cases with Rscript. This pipeline is applicable only to HIM-mediated duplications for which both extremities are identified and for which we can obtain the junctions. Thus, we were able to look for shared HIM-mediated duplications for 5 segments, i.e., S16_Hdp, S10_Hdp1, S10_Hdp2, S26_Hdp1, and S26_Hdp2. We also searched for additional candidate HIM-mediated duplications that would be specific to each genome. For this, we used the result of the first BLASTN output and that of a BLASTN similarity search between *Cotesia* genomes and DRJs (same options as for the two first searches). This third output allowed us to identify cases in which the 5′ DRJ and the 3′ DRJ of the same segment aligned next to each other (and not at the extremities of the segments, in contrast to proviral segments), as expected for HIM-mediated integrations (30).

### Sequencing of S. nonagrioides larvae parasitized by C. typhae.

C. typhae individuals used for this experiment were taken from the strain of Kobodo coming from International Centre of Insect Physiology and Ecology rearing (see “DNA extraction, library preparation, and sequencing of the C. typhae genome”) and reared at EGCE with a protocol set up to limit inbreeding. S. nonagrioides larvae came from a strain reared at EGCE since 2010 from individuals collected in several localities in southwest France and refreshed yearly with such individuals. Eighteen S. nonagrioides larvae were each parasitized by a different C. typhae female. Ovipositions were confirmed by visual observations for all of them. During oviposition, C. typhae lays a relatively large number of eggs in its host, generally ranging between 70 and 110 eggs (34). Larval development typically takes about 14 days under laboratory conditions until wasp larvae emerge from their host and pupate (52). Here, we placed the larvae at −80°C 7 days after oviposition. We then dissected the 18 larvae to check for the presence of wasp larvae, which at this stage measure about 5 mm and can be easily spotted by eye. The apparent success of wasp larval development before their storage was observed in 12 caterpillars. We then collected hemolymph, heads, ganglionic chains, and fat bodies from 6, 3, 9, and 1, respectively, of these 12 caterpillars. In total, we collected 780 μl of hemolymph. The minimum amount of each tissue necessary to extract sufficient amounts of DNA for Illumina sequencing (at least 500 ng at a concentration of at least 50 ng/μl) was determined in a separate experiment. Except for the hemolymph, all samples were rinsed multiple times with phosphate-buffered saline. DNA was then extracted from a pool of each tissue (except the fat body) using the DNeasy blood and tissue kit (Qiagen). We also extracted DNA from 1 of the 6 whole larvae in which we were unable to find any wasp embryos. We subcontracted Novogen to build a paired-end library (2 × 150 bp; insert size, 350 bp) for each sample. Each sample was then sequenced on an Illumina platform to produce a targeted amount of 100 Gbp.

### Assessment of sequencing coverage on the genome of C. typhae and S. nonagrioides.

Sequencing coverage was assessed on the genome of C. typhae assembled in this study, as well as on that of S. nonagrioides described by Muller et al. (40) (GenBank accession number JADWQK000000000). In brief, the genome was assembled using short Illumina reads and long ONT reads using the MaSurCA assembler (81), followed by a run of the purge_dup pipeline (82) to remove scaffolds with low coverage, partial overlaps, and haplotigs. The resulting assembly is composed of 2,253 scaffolds with an *N*_50_ value of 1,105 kbp and a total size of 1,021 Mpb. It contains 96% of Lepidoptera BUSCO genes, 2.7% of which are duplicated (40).

Adapters were removed and reads were quality trimmed with Trimmomatic v0.38 (options LEADING:20, TRAILING:20, SLIDINGWINDOW:4:15, and MINLEN:36) (83). Raw and trimmed read quality was assessed using FastQC v0.11.8 (84). To obtain statistics on sequencing depth, we aligned trimmed paired-end reads from the 5 samples using Bowtie2 v2.3.4.2 in end-to-end mode separately on the wasp and moth genomes (85). The resulting SAM files were sorted and converted into BAM files with SAMtools v1.7. Finally, sequencing depth was calculated with bedtools genomecov v2.26.0 for each sample for both C. typhae and S. nonagrioides genomes.

### Characterization of CtBV circle integrations into the genome of S. nonagrioides.

Raw fastq files were converted into fasta files with the seqtk seq command (option -a). Resulting fasta files were aligned on the C. typhae genome with BLASTN v2.6.0 (options -task megablast, -max_target_seqs 2 -outfmt 6). Reads that aligned on C. typhae were extracted and aligned on the S. nonagrioides genome, with the same options. The resulting outputs contained alignment coordinates and other information for each read aligning on both reference genomes.

We used these outputs to identify integrations of CtBV DNA circles throughout the S. nonagrioides genome. For that, we searched for sequencing reads for which a portion aligned on the S. nonagrioides genome only and the other portion aligned on CtBV proviral segments only. Such chimeric reads were identified using an R pipeline that was previously used to identify recombination events within a single genome and that we slightly adapted for our study (86). After this pipeline, we filtered out the PCR duplicates. Briefly, wasp-caterpillar chimeric reads are identified based on the tabular BLASTN outputs as follows: (i) at least 16 bases must align only on C. typhae, and a minimum of 16 other bases must align only on S. nonagrioides; (ii) less than 10% of the read length is allowed to map to neither reference genome; (iii) no more than 20 bases can align simultaneously on both reference genomes; and (iv) no more than 5 bases must be inserted between the two genomes at the integration point. The two latter filters imply that aligned read regions are allowed to overlap by up to 20 bp or to be separated by at most 5 bp. The overlap corresponds to microhomology between CtBV DNA circles and the host genome at the integration point, whereas the separation corresponds to nontemplated addition of nucleotides at the integration point (86, 87). To check whether the microhomology lengths at integration points were consistent with those expected by chance, we simulated expected distributions following the approach described by Peccoud et al. (86). Briefly, considering the sequences of the CtBV circles and the S. nonagrioides genome, the distribution of homology lengths was compared to that of random chimeric reads generated *in silico*. Each *in silico* read was made of two regions extracted from random locations of CtBV circles and the S. nonagrioides genome. The lengths of the two regions were chosen at random, with the conditions that both were at least 28 bp and their sum was the size of a read (150 bp). These reads were then subjected to a BLAST search against the sequences from which they were generated, and the BLAST outputs were subjected to the same analysis as that performed on real data.

### Localization of chimeric reads in CtBV circles.

Chimeric reads mapping to CtBV circles were assigned to three categories depending on the position of the wasp-host junction, i.e., (i) chimeric reads for which the CtBV-host junction falls within HIMs, (ii) reads for which the junction falls in circles devoid of HIMs or outside HIMs in circles containing HIMs, and (iii) reads for which the wasp-host junction falls precisely in the J1 and J2 regions. The last category is included in the first one. The J1 and J2 regions were defined as the positions supported by the most chimeric reads plus the positions around that point until a position was supported by <2 reads. We defined J1 and J2 independently for each sample. To assess whether integration not involving HIMs was specific to bracovirus circles or whether it also occurred for any wasp genome regions, we compared the number of chimeras falling outside HIMs in bracovirus circles to those found in exons of wasp BUSCO genes. Considering the length and sequencing depth of BUSCO gene exons and bracovirus circles, we calculated an expected number of chimeric reads for each circle in each sample. We then compared these expected numbers to the observed numbers of chimeras falling outside HIMs.

### Data availability.

The assembly and annotation of the C. typhae genome are available in GenBank under the accession number JAAOIC000000000.2 and at the BioInformatics Platform for Agroecosystem Arthropods (BIPAA) (https://bipaa.genouest.org/sp/cotesia_typhae/). The raw sequencing reads for the 5 samples of S. nonagrioides parasitized by C. typhae are available in the NCBI database under BioProject number PRJNA718433.
