# Supplementary material for: Genome-Wide Patterns of Bracovirus Chromosomal Integration into Multiple Host Tissues during Parasitism
Source: J Virol. 2021 Oct 27;95(22):e00684-21. doi: 10.1128/JVI.00684-21 (PMC8549517; doi:10.1128/JVI.00684-21)
Supplement: Supplemental file 1 — Tables S1 to S6; Fig. S1 to S6. Download JVI.00684-21-s0001.pdf, PDF file, 6.1 MB [file jvi.00684-21-s0001.pdf]

Table S1: Summary of sequenced reads for *C. typhae*'s genome assembly

Sequencer	# reads	#Bases	#seq. > 10kb	#seq. > 30kb	#seq. > 50kb	#seq. > 100kb	Mean quality*	Accession number
MiSeq	17 050 964	9 122 372 981						SRR11496006
MinION	2 313 105	4 975 381 422	68 874	5 482	602	4	12.92	SRR11496005
MinION	618 267	8 682 728 038	345 696	60 512	6 852	107	14.56	SRR11496004
PromethION	5 540 364	56 119 510 441	2 158 624	363 664	39 432	599	15.56	SRR11496003

*Average quality score (Q score) of the long reads

Table S2: *C. typhae*'s *a*ssembly statistics

Assembly method	#scaffolds	Total size (bp)	N50 scaffold length (bp)	Longest scaffold length (bp)
Short Illumina assembly	262 110	182 687 155	5 012	143 403
ONT assembly	86	190 664 434	6 968 763	20 425 376
Polished ONT assembly	72	186 662 351	6 806 882	19 983 142

Table S3: Summary of BUSCO results on *C. typhae*'s *genome*

	**Retained *C. typhae* assembly**
Complete BUSCOs	1623 (97.9%)
Complete and single-copy BUSCOs (S)	1606 (96.9%)
Complete and duplicated BUSCOs (D)	17 (1.0%)
Fragmented BUSCOs (F)	16 (1.0%)
Missing BUSCOs (M)	19 (1.1%)
Total BUSCO groups searched	1658 (100%)

Table S4: *C. typhae*'s annotation statistics

**Type of annotated element**	**Number**
Number of genes	8591
Number of mrnas	8597
Number of mrnas with utr both sides	1399
Number of mrnas with at least one utr	4106
Number of cdss	8597
Number of exons	45775
Number of five_prime_utrs	2964
Number of three_prime_utrs	2541
Number of exon in cds	42634

Number of exon in five_prime_utr	4758
Number of exon in three_prime_utr	3854
Number of intron in cds	34037
Number of intron in exon	37178
Number of intron in five_prime_utr	1794
Number of intron in three_prime_utr	1313
Number gene overlapping	181
Number of single exon gene	404
Number of single exon mrna	405
mean mrnas per gene	1
mean cdss per mrna	1
mean exons per mrna	5,3
mean five_prime_utrs per mrna	0,3
mean three_prime_utrs per mrna	0,3
mean exons per cds	5
mean exons per five_prime_utr	1,6
mean exons per three_prime_utr	1,5
mean introns in cdss per mrna	4
mean introns in exons per mrna	4,3
mean introns in five_prime_utrs per mrna	0,2
mean introns in three_prime_utrs per mrna	0,2
Total gene length	37213247
Total mrna length	37225622
Total cds length	12782792
Total exon length	14738041
Total five_prime_utr length	861486
Total three_prime_utr length	1093763
Total intron length per cds	20408156
Total intron length per exon	22524759
Total intron length per five_prime_utr	1421292
Total intron length per three_prime_utr	665082
mean gene length	4331

mean mrna length	4330
mean cds length	1486
mean exon length	321
mean five_prime_utr length	290
mean three_prime_utr length	430
mean cds piece length	299
mean five_prime_utr piece length	181
mean three_prime_utr piece length	283
mean intron in cds length	599
mean intron in exon length	605
mean intron in five_prime_utr length	792
mean intron in three_prime_utr length	506
Longest gene	154568
Longest mrna	154568
Longest cds	26061
Longest exon	13665
Longest five_prime_utr	14754
Longest three_prime_utr	14292
Longest cds piece	13665
Longest five_prime_utr piece	7168
Longest three_prime_utr piece	6076
Longest intron into cds part	73685
Longest intron into exon part	73685
Longest intron into five_prime_utr part	10629
Longest intron into three_prime_utr part	41977
Shortest gene	138
Shortest mrna	138
Shortest cds	36
Shortest exon	2
Shortest five_prime_utr	1
Shortest three_prime_utr	1
Shortest cds piece	1

Shortest five_prime_utr piece	1
Shortest three_prime_utr piece	1
Shortest intron into cds part	1
Shortest intron into exon part	2
Shortest intron into five_prime_utr part	11
Shortest intron into three_prime_utr part	1

Table S5: Coordinates of the 28 proviral segments in C. typhae with the positions of their DRJs and HIMs. The columns 'Start' and 'End 'indicate the coordinates of the proviral segments and 'Orientation' indicates whether the segment is oriented from DRJ5 to DRJ3 (+) in the reference genome or from DRJ3 to DRJ5 (-). 'DRJ1_end' indicates the end of the first DRJ (DRJ5 or DRJ3 depending on the orientation), the beginning being the same coordinate than 'Start', whereas 'DRJ2_start' indicates the start of the other DRJ, the end being the same coordinate than 'End'. HIM_start' and 'HIM_end' indicate the coordinates of the HIMs for the segments with HIMs, the others have NA. The coordinates of the segments in red and orange in Figure A have to be taken with caution because we were not able to identify one of the DRJ (NA in the table).

Segment	Contig	Start	End	DRJ1_end	DRJ2_start	HIM_start	HIM_end	Orientation
S1	contig_4	10779988	10800059	10799953	10780048	10780296	10780417	-
S10	scaffold_91	1058754	1076122	1058847	1076019	1075732	1075854	+
S11	contig_26	1347572	1359376	1359316	1347679	1347840	1347970	-
S12	contig_26	1359742	1373010	1359848	1372887	1359880	1359997	+
S14	contig_26	1317007	1345253	NA	1317201	1317405	1317525	-
Ps15	contig_36	30332	30658	NA	NA	NA	NA	+
S16	contig_36	124144	133421	124219	133311	132487	132609	+
S17	scaffold_91	1076379	1090049	1089925	1076438	1076603	1076725	-
S18	contig_36	98856	123006	99056	122900	122749	122854	-
S19	contig_69	70049	76798	70162	76595	NA	NA	+
S2	contig_276	13828	29878	14025	29824	NA	NA	-
S20/33	contig_276	29896	54070	29896	54006	NA	NA	+
S23	contig_276	163165	166344	163338	NA	NA	NA	-
S24	contig_36	64670	85950	85868	NA	85710	85818	-
S25	contig_69	1	69858	69751	NA	NA	NA	-
S26	contig_4	11078861	11088063	11087876	11078937	11087734	11087858	-
S27	contig_36	1	30242	75	30134	30006	30122	-
S28	contig_323	4514	41675	41571	4622	41301	41439	-
S30	contig_246	1	10520	88	NA	NA	NA	+
S32	contig_36	30722	63950	30825	63856	63655	63779	+
S35	contig_36	86101	98819	86208	98722	90702	90835	+
S36	contig_276	213	13813	317	13716	NA	NA	+
S37	contig_323	667	4356	NA	4253	NA	NA	+
S4	scaffold_91	989293	998487	989406	998361	998131	998251	+
S5	contig_276	143626	151685	143725	151494	NA	NA	+
S6	contig_276	153972	162773	154098	162622	NA	NA	-
S7	contig_36	355273	373607	373502	355427	355747	355880	-

Table S6: Number of chimeric reads involving CtBV circles in each sample

	Whole body	Hemolymph	Fat body	Ganglionic chain	Head
CtBV/*S.nonagrioides* chimera	730	3126	1015	1158	1083
S. nonagrioides trimmed reads	310568520	245108456	547510065	308813876	342540175
Relative chimeric reads in IPMH*	2,350527993	12,75353797	1,853847198	3,7498315	3,161672934

* IPMH stands for IE per million reads mapping on the host (*S. nonagrioides*). It is calculated as follow:
*(row 1 / row 2) * 10^6*

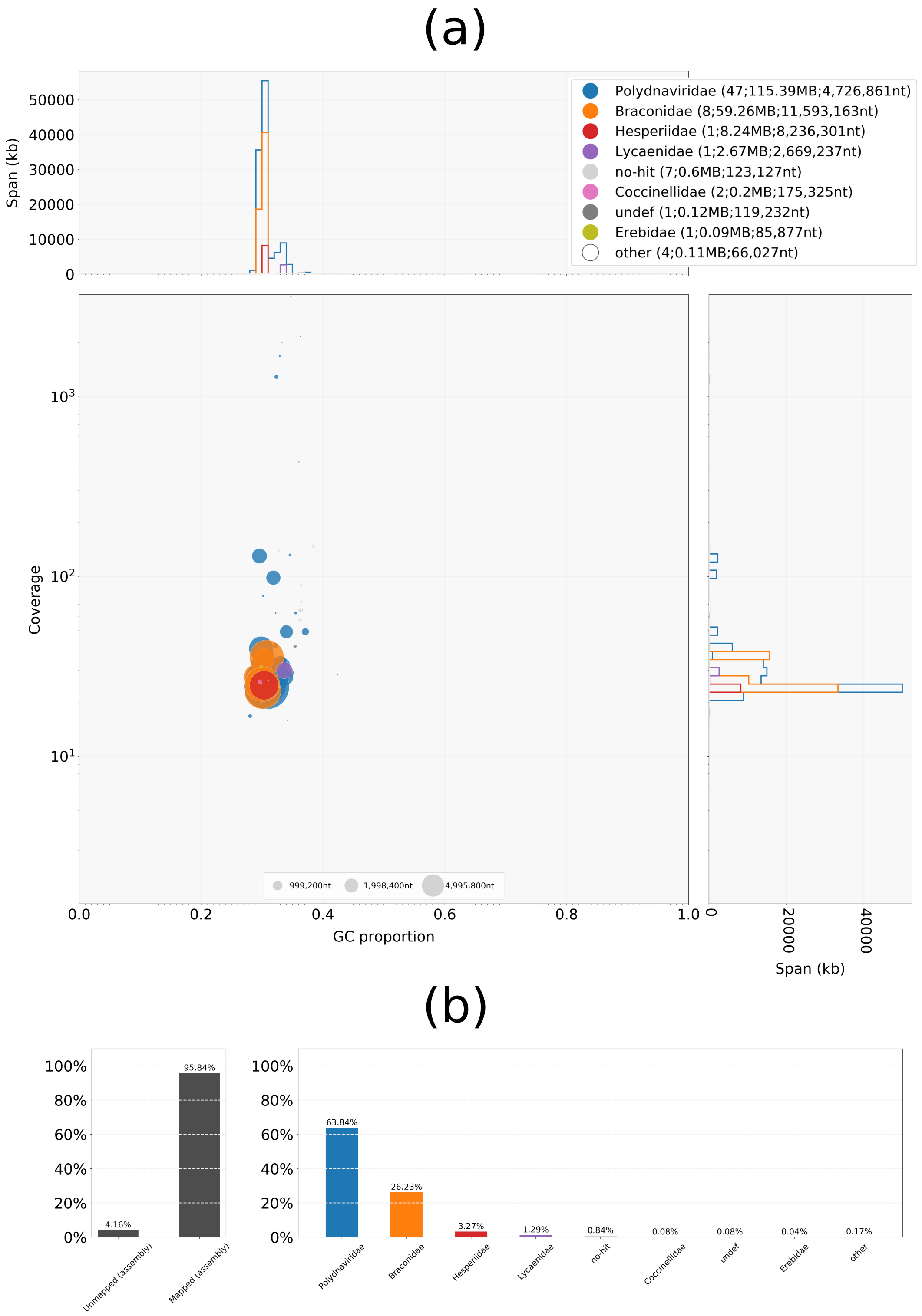

Figure S1. Assessment of contamination in *Cotesia typhae* genome assembly. (a) Blobplot in which each circle represents a contig. Circle's diameters are scaled proportionally to scaffold length and colored according to taxonomic affiliation. (b) ReadCovPlots visualizing the percentage of reads mapping on each taxonomic affiliation.

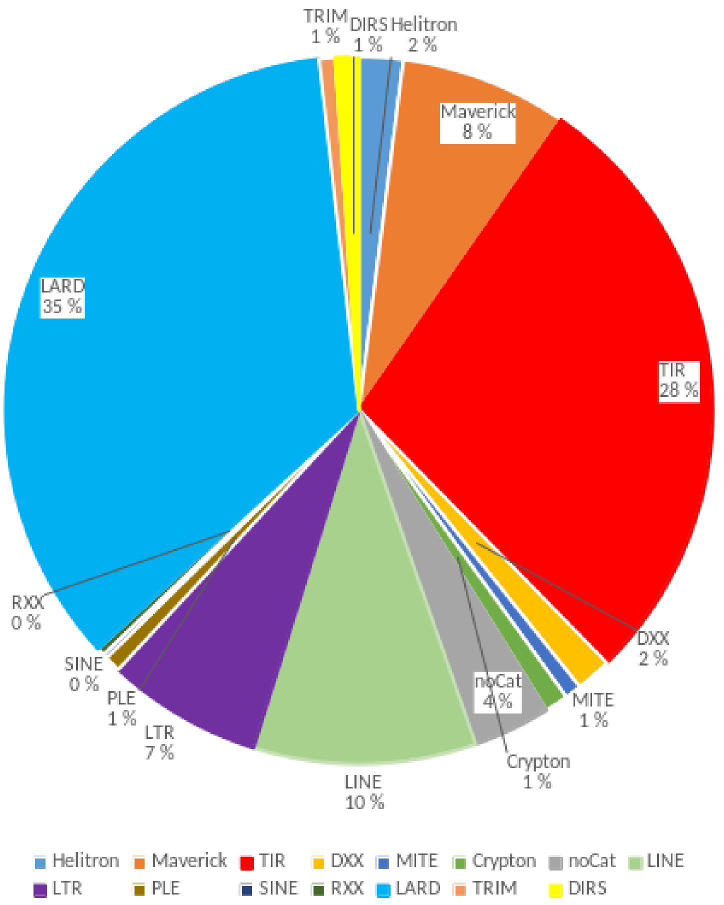

Figure S2. Transposable element composition in *C. typhae* genome. Each color represents a TE family, whose name and percentage are indicated in the pie.

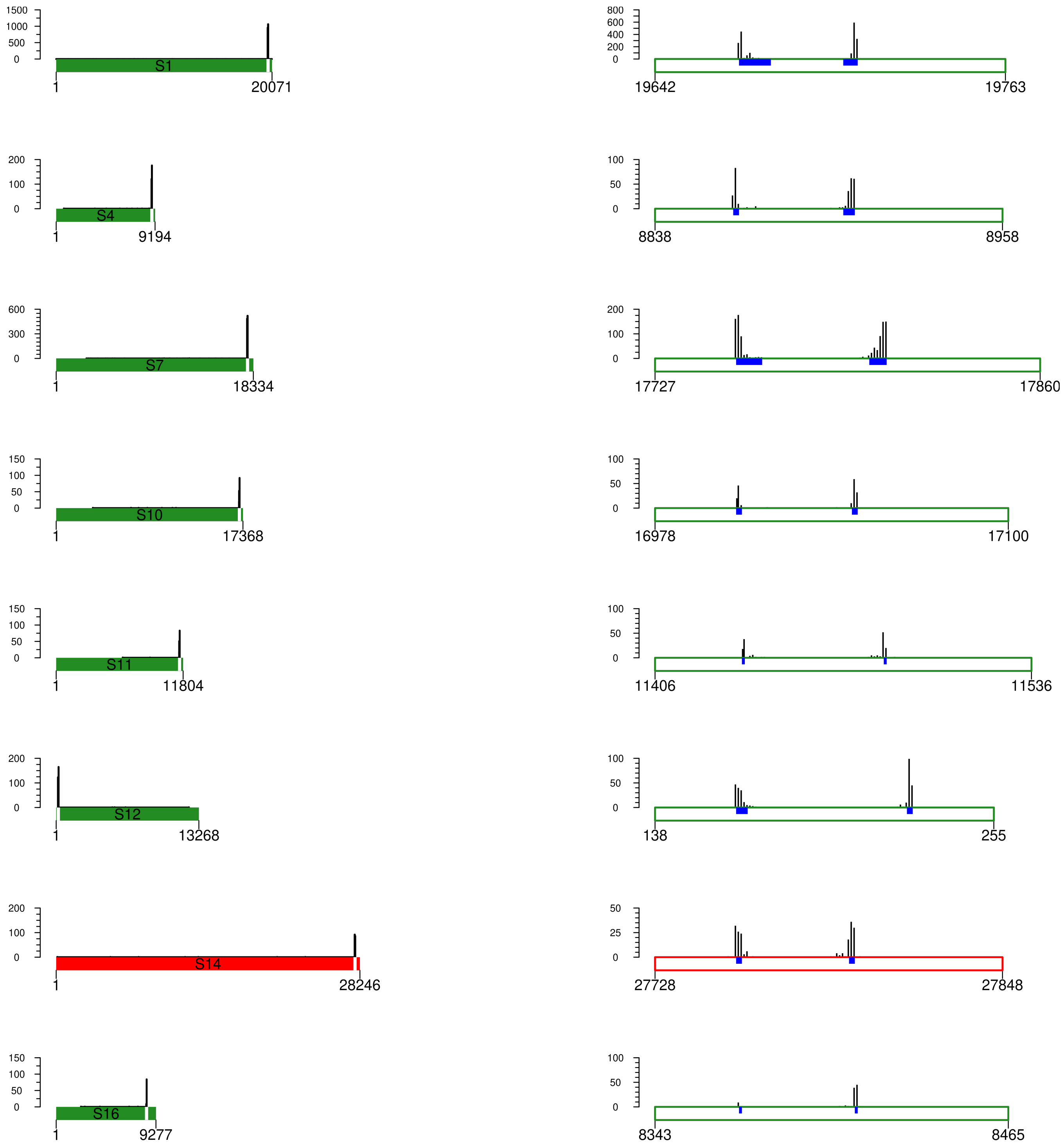
1500
1000
500
0
S1
1
20071
800
600
400
200
0
19642
19763
200
100
0
S4
1
9194
100
50
0
8838
8958
600
300
0
S7
1
18334
200
100
0
17727
17860
150
100
50
0
S10
1
17368
100
50
0
16978
17100
150
100
50
0
S11
1
11804
100
50
0
11406
11536
200
100
0
S12
1
13268
100
50
0
138
255
200
100
0
S14
1
28246
50
25
0
27728
27848
150
100
50
0
S16
1
9277
100
50
0
8343
8465

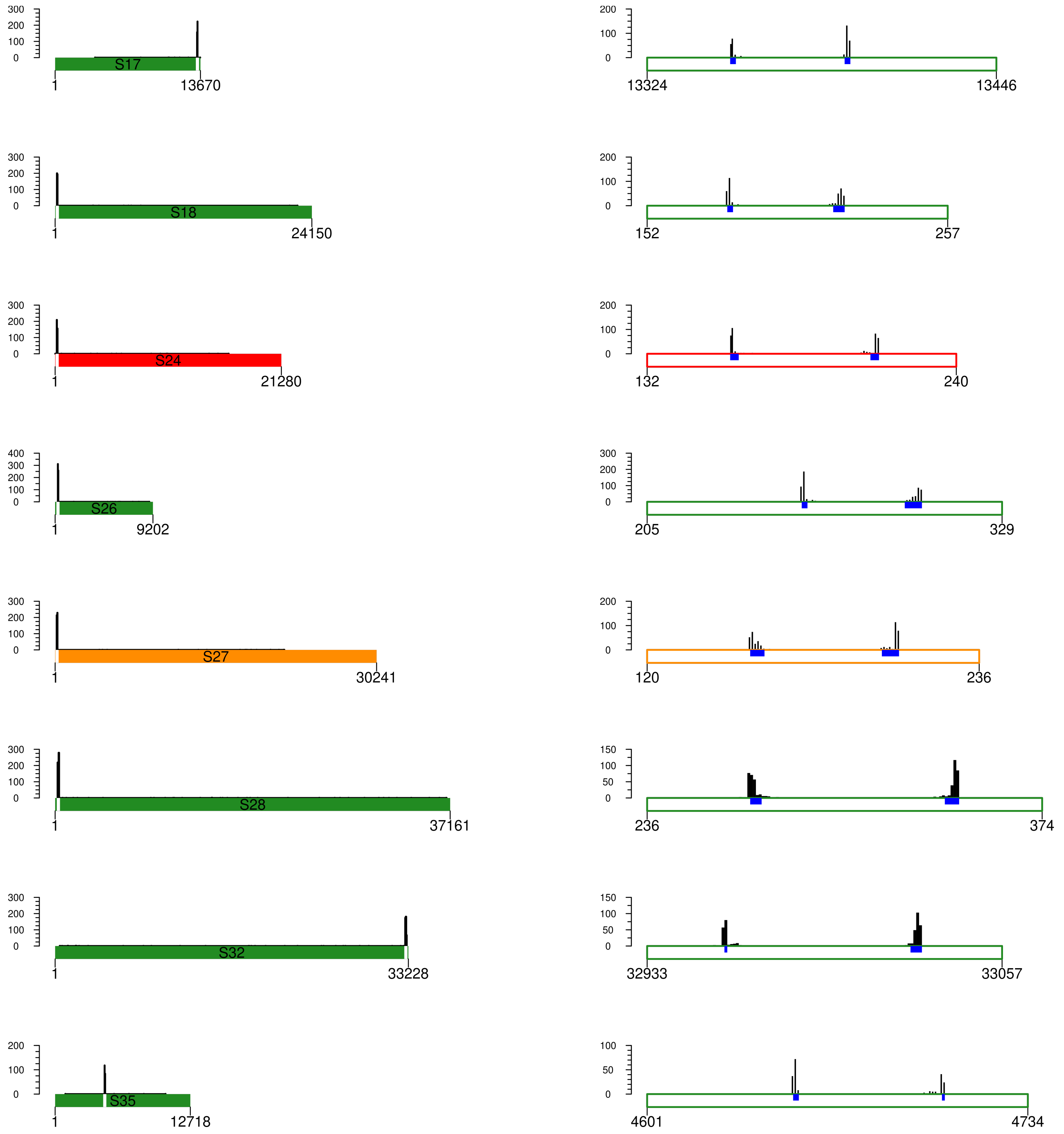

Figure S3. Landscape of the chimeric points in the 16 proviral segments with HIMs. The histograms on the left show the whole segments, where HIMs are indicated in white. In order to be visible, the white region is 300 bp, although the HIMs are between 105 and 138 bp-long. Under the segments, we indicated the position 1 and the last position of each segment. The names of the segments are indicated in the coloured rectangles. The colours are in accordance with Figure 1, where they represent the quality of the annotation of the segments. Most chimeric reads are located in the HIMs. The chimeric reads outside HIMs are split all over the segments. The y axis represents the number of

chimeric reads by bin, summed up for all samples. The x axis are in scale between segments. The histograms on the right are zooms of HIMs. We indicated the coordinates of the HIMs. The x axis are also in scale between segments. In the HIMs, chimeric reads are split into two regions, called J2 for the left one and J1 for the right one. J2 and J1 positions were defined as the position supported by the most chimeric reads for each peak, plus the positions around this point until a position is supported by less than two reads. This was done independently for each sample. All histograms are oriented from DRJ5 to DRJ3.

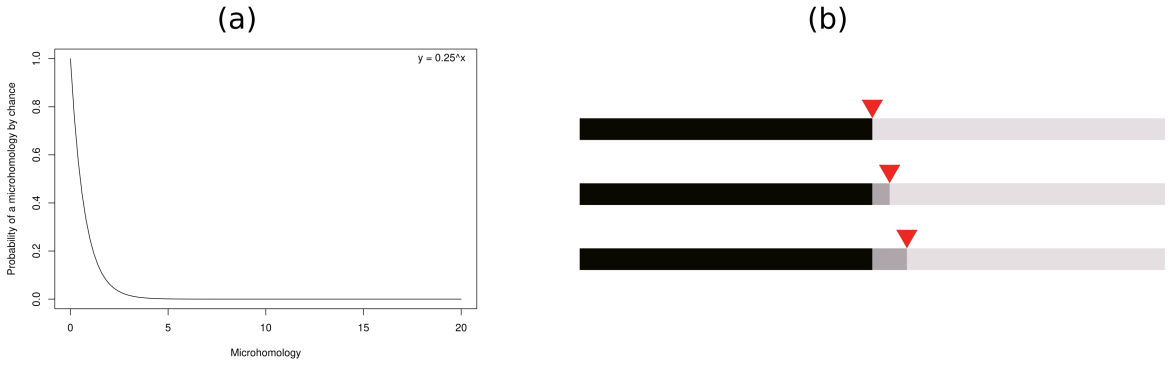

Figure S4. Illustration of microhomologies in chimeric reads. (a) Probability of a microhomology between two DNA sequences. The maximum possible microhomology is 20 because a chimeric read with a higher overlap between the two species is not kept by our pipeline. (b) Identification of the chimeric point by our pipeline. Three chimeric reads are shown, with in black the CtBV DNA, in light gray the caterpillar DNA and in dark gray the region aligning on both CtBV and caterpillar sequence (microhomology). The red triangles correspond to the coordinate that is considered as the chimeric point in CtBV by our pipeline. Regardless of the microhomology, the pipeline always associates the last coordinates of CtBV as the chimeric point.

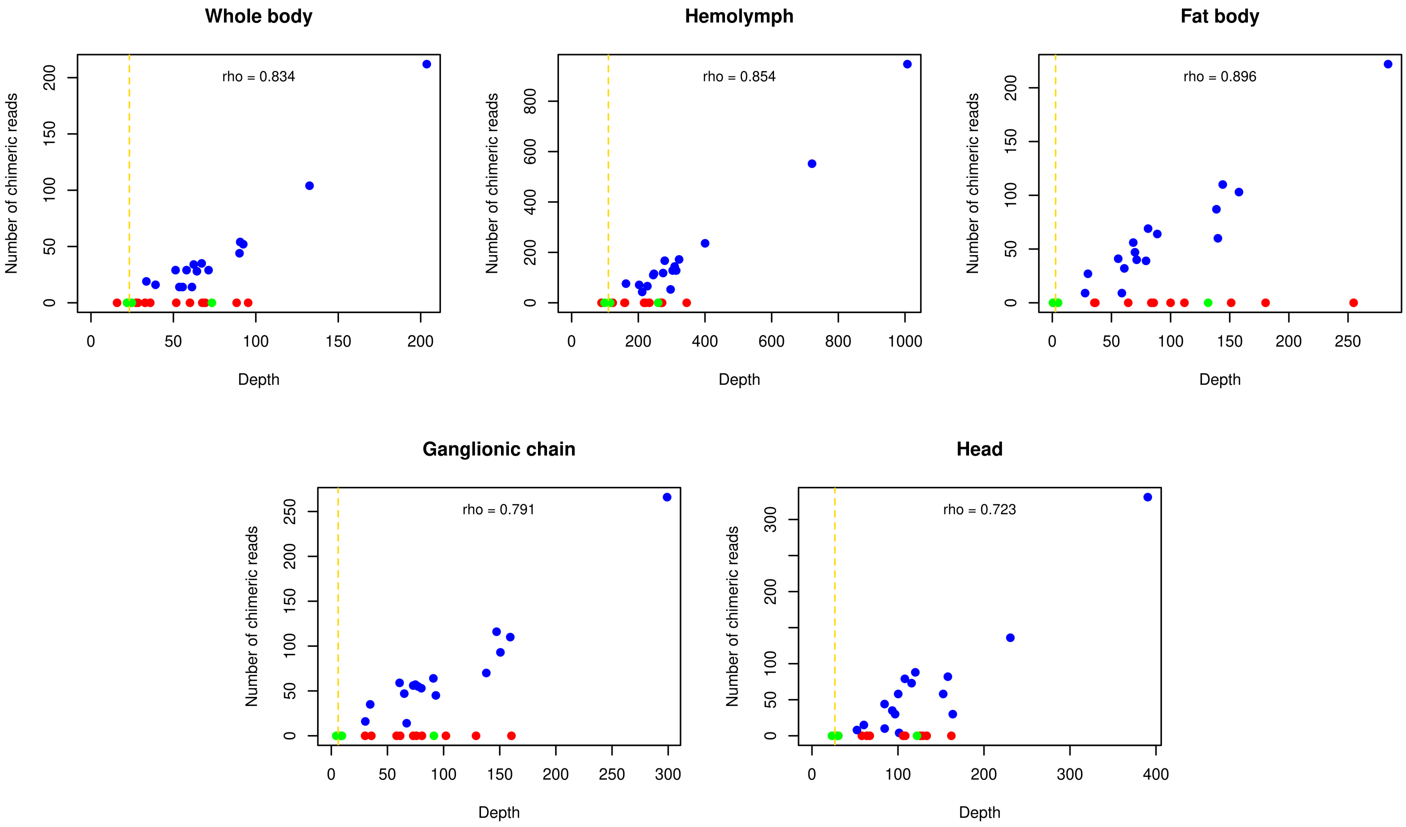

Figure S5. Relationships between sequencing depth and number of chimeric reads for each segment and sample. For each sample, blue dots represent proviral segments with HIM-mediated integrations, while red dots represent proviral segments without such integrations. Green dots represents duplicated segments, ie Rdp and Hdp. The yellow dash line shows the average coverage on *C. typhae* genome in the sample. Only proviral segments with HIM-mediated integrations are taken into account to calculate the rho of Sperman, showing the correlation between sequencing depth and number of IEs.

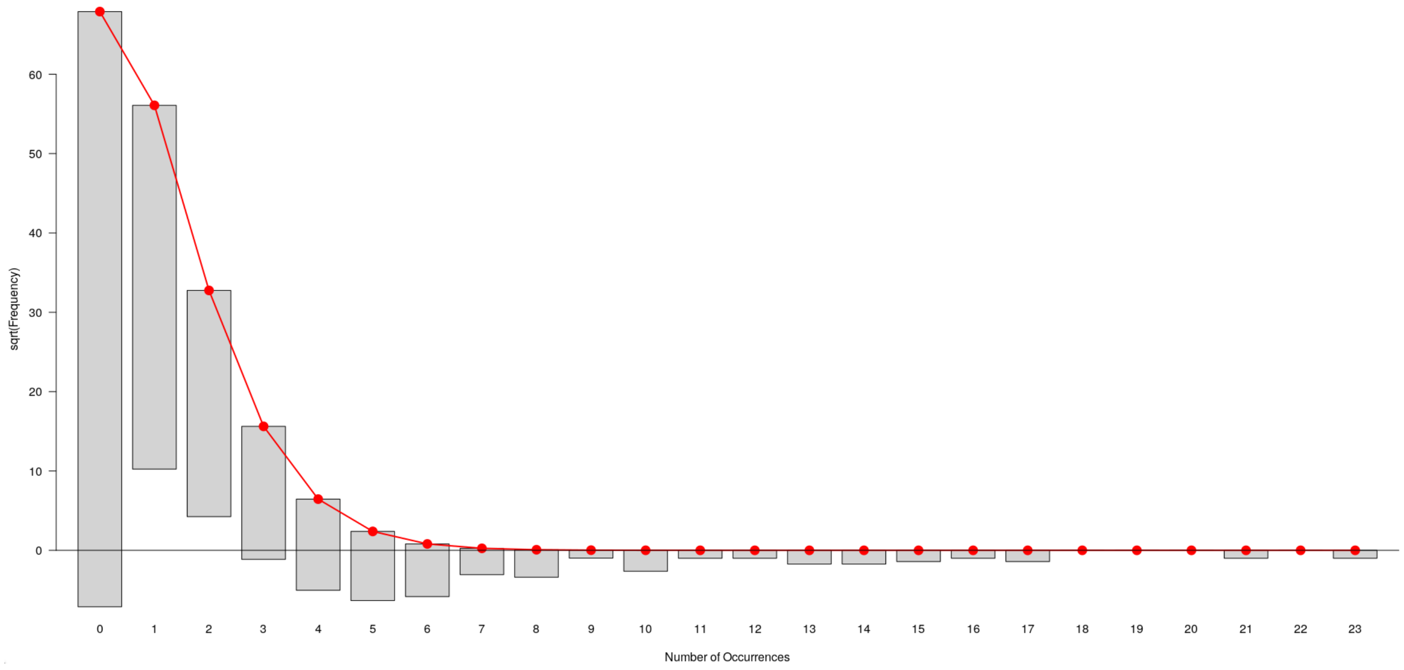

Figure S6. Number of 100,000 bp windows with a certain amount of IE. Because of visual constraints, we showed the amount of IE as the squared root of the number of IE. We summed up the number of IE for all samples. Observed values are represented by the gray barplot and expected values under a poisson distribution are represented in red. Bars for which the bottom is above the x axis indicates that the observed value is under-represented whereas bars for which the bottom is under the x axis indicates that the observed value is over-represented.
